# Dual molecular bridges at perovskite heterointerfaces for efficient inverted solar cells

**DOI:** 10.1093/nsr/nwaf211

**Published:** 2025-05-22

**Authors:** Qing Lian, Lina Wang, Guoliang Wang, Guojun Mi, Bowei Li, Joel A Smith, Pietro Caprioglio, Manuel Kober-Czerny, Deng Wang, Qiming Yin, Jiong Yang, Sibo Li, Xiao Liang, Shaokuan Gong, Dongyang Li, Hanlin Hu, Xihan Chen, Xugang Guo, Longbin Qiu, Baomin Xu, Gang Li, Anita W Y Ho-Baillie, Wei Zhang, Guangfu Luo, Henry J Snaith, Chun Cheng

**Affiliations:** Department of Materials Science and Engineering, Southern University of Science and Technology, Shenzhen 518055, China; Department of Materials Science and Engineering, Southern University of Science and Technology, Shenzhen 518055, China; Department of Materials Science and Engineering, Southern University of Science and Technology, Shenzhen 518055, China; School of Physics and The University of Sydney Nano Institute, The University of Sydney, Sydney, NSW 2006, Australia; Department of Materials Science and Engineering, Southern University of Science and Technology, Shenzhen 518055, China; Future Photovoltaic Research Center, Global Institute of Future Technology, Shanghai Jiao Tong University, Shanghai 200240, China; Clarendon Laboratory, Department of Physics, University of Oxford, Oxford OX1 3PU, UK; Clarendon Laboratory, Department of Physics, University of Oxford, Oxford OX1 3PU, UK; Clarendon Laboratory, Department of Physics, University of Oxford, Oxford OX1 3PU, UK; Department of Materials Science and Engineering, Southern University of Science and Technology, Shenzhen 518055, China; Department of Materials Science and Engineering, Southern University of Science and Technology, Shenzhen 518055, China; Department of Chemistry, Southern University of Science and Technology, Shenzhen 518055, China; SUSTech Energy Institute for Carbon Neutrality, Department of Mechanical and Energy Engineering, Southern University of Science and Technology, Shenzhen 518055, China; Hoffman Institute of Advanced Materials, Shenzhen Polytechnic, Shenzhen 518055, China; SUSTech Energy Institute for Carbon Neutrality, Department of Mechanical and Energy Engineering, Southern University of Science and Technology, Shenzhen 518055, China; Department of Materials Science and Engineering, Southern University of Science and Technology, Shenzhen 518055, China; Department of Electronic and Information Engineering, Research Institute for Smart Energy, The Hong Kong Polytechnic University, Hong Kong 999077, China; Hoffman Institute of Advanced Materials, Shenzhen Polytechnic, Shenzhen 518055, China; SUSTech Energy Institute for Carbon Neutrality, Department of Mechanical and Energy Engineering, Southern University of Science and Technology, Shenzhen 518055, China; Department of Materials Science and Engineering, Southern University of Science and Technology, Shenzhen 518055, China; SUSTech Energy Institute for Carbon Neutrality, Department of Mechanical and Energy Engineering, Southern University of Science and Technology, Shenzhen 518055, China; Department of Materials Science and Engineering, Southern University of Science and Technology, Shenzhen 518055, China; Department of Electronic and Information Engineering, Research Institute for Smart Energy, The Hong Kong Polytechnic University, Hong Kong 999077, China; School of Physics and The University of Sydney Nano Institute, The University of Sydney, Sydney, NSW 2006, Australia; Advanced Technology Institute, Department of Electrical and Electronic Engineering, University of Surrey, Guildford GU2 7XH, UK; State Centre for International Cooperation on Designer Low-carbon & Environmental Materials (CDLCEM), School of Materials Science and Engineering, Zhengzhou University, Zhengzhou 450001, China; Department of Materials Science and Engineering, Southern University of Science and Technology, Shenzhen 518055, China; Clarendon Laboratory, Department of Physics, University of Oxford, Oxford OX1 3PU, UK; Department of Materials Science and Engineering, Southern University of Science and Technology, Shenzhen 518055, China; SUSTech Energy Institute for Carbon Neutrality, Department of Mechanical and Energy Engineering, Southern University of Science and Technology, Shenzhen 518055, China; Guangdong Provincial Key Laboratory of Energy Materials for Electric Power, Southern University of Science and Technology, Shenzhen 518055, China

**Keywords:** perovskite solar cell, molecular bridge, heterointerface engineering, self-assemble material

## Abstract

Utilizing molecular bridges presents a promising means to enhance the performance of perovskite solar cells (PSCs). However, concurrently bridging the perovskite absorber and its two adjacent interfaces remains a significant challenge that is yet to be achieved. Here, we construct dual molecular bridges at perovskite heterointerfaces, enabled by a self-organizing additive of 4-fluoro-phenethylammonium formate (4-F-PEAFa) and a synthesized hole transporter of [2-(7H-dibenzo[c, g]carbazol-7-yl)ethyl]phosphonic acid (DBZ-2PACz). The molecular bridges spanning two interfaces lead to the formation of an ‘integral carrier transport pathway’, mitigating both non-radiative recombination and charge-transport losses in the fabricated PSC devices. We thus achieve a champion power conversion efficiency (PCE) of 26.0% (25.6% certified) in inverted PSCs, accompanied by an exceptionally high fill factor of 0.87 (maximum 0.88 from the certified devices, 97% of its Shockley–Queisser limit) and a low ideality factor of 1.06. The unencapsulated devices retain 96% of their PCEs after aging at 85°C for 2200 h and 90% after maximum power point tracking at an elevated temperature of 50°C for 973 h.

## Teaser

Dual interfacial bridges enable efficient charge transport in inverted perovskite solar cells, yielding 25.6% certified efficiency and a fill factor exceeding 0.87.

## INTRODUCTION

Considering the rapid growth of power conversion efficiencies (PCEs), e.g. from 3.9% in 2009 to 27.0% in 2025 [1,2], metal halide perovskite solar cells (PSCs) have shown great potential amongst the next-generation thin-film photovoltaics, in which the device normally consists of an n-i-p or p-i-n structure (also called inverted). Inverted PSCs (IPSCs) were first created in 2013 [3], standing out as promising contenders recently due to their fascinating attributes, such as simplified structure, low-temperature fabrication, excellent operational stability, diverse choices of charge transport materials, and compatibility with flexible substrates, multijunction cells and large-scale production [4–6]. However, most high-performance IPSCs (e.g. with certified PCEs >25%) still exhibit suboptimal fill factors (FFs, more details can be found in Table S1), primarily due to the interplay between charge recombination and extraction processes [7–12].

The continued advancement of PCE relies on substantial improvement in FF whilst concurrently upholding high open-circuit voltage (*V*_OC_) and short-circuit current density (*J*_SC_). This requires collective efforts to address the imperfect perovskite crystallization and the non-ideal adjacent interfaces, namely the buried interface between the perovskite and hole transport layer and the top interface between the perovskite and electron transport layer, both of which are closely linked with the notorious non-radiative recombination and charge-transport losses [13]. To tackle these challenges, perovskite crystallization engineering and interface modifications are commonly implemented [14–18], including precise control of reaction kinetics [16,17] and the incorporation of multiple additives and treatments [18]. Despite their effectiveness in passivating defects and reducing recombination losses to enhance the *V*_OC_, these techniques often fail to leverage this passivation effect for improved charge extraction. Additionally, while such approaches could provide a modest improvement in FF, they are insufficient to mitigate charge-transport losses due to the inevitable increase in contact resistance. Recent studies indicate that constructing carrier viaducts through molecular bridging is an efficient strategy for charge extraction and transport [19,20]. However, this approach is largely confined to improving an individual functional layer or specific interface instead of the entire device. Thus, it is imperative to develop a holistic approach to enable the fabrication of high-performance IPSCs with excellent reproducibility and long-term stability.

In this study, we construct dual molecular bridges at perovskite adjacent heterointerfaces through the combination of the additive (4-fluoro-phenethylammonium formate, 4-F-PEAFa) and hole transporter ([2-(7H-dibenzo[c, g]carbazol-7-yl)ethyl]phosphonic acid, DBZ-2PACz). This strategy enables the comprehensive regulation of perovskite absorber and its adjacent interfaces, resulting in both low non-radiative recombination losses and carrier transport losses. Based on a series of theoretical and experimental characterizations, we demonstrate that 4-F-PEAFa can spontaneously distribute throughout the as-crystalized perovskite film, increasing the grain size, charge carrier mobilities, and quasi-Fermi level splitting when in contact with both charge transport layers. Thanks to the self-organizing characteristic, 4-F-PEAFa firmly bonds with the perovskite surface and bridge both the bottom hole transporter and top electron transporter, leading to the formation of ‘dual molecular bridges’ throughout IPSC devices. This integral carrier transport pathway enhances carrier extraction/transport and thus substantially increases the device FF. Coupled with our synthesized DBZ-2PACz hole transporter, we realize IPSCs with a certified champion PCE of 25.6% and FF of 0.87 (with a certified maximum of 0.88 and a low ideality factor of 1.06), approaching its Shockley–Queisser limit at the corresponding bandgap. Encouragingly, the long-term stability of devices has shown significant improvement under three different levels of the international Summit on Organic Photovoltaic Stability (ISOS) protocols, including ISOS-D-1, ISOS-D-2I, and ISOS-L-2I testing.

## RESULTS AND DISCUSSION

### Holistic construction of molecular bridges

**Figure 1.**
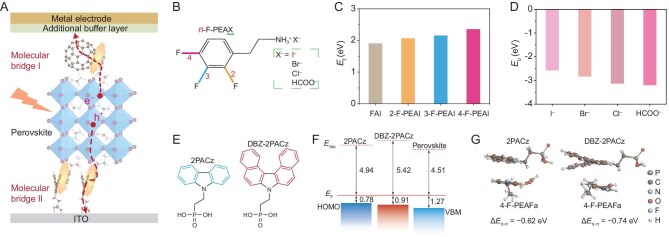
Construction of molecular bridges at perovskite adjacent interfaces. (A) Schematic of the proposed approach in the entire device. The yellow ellipse and red dash lines represent the interaction and the carrier pathway, respectively. (B) Cation and anion engineering of *n*-F-PEAX. ‘*n*’ denotes the substitutional position of the fluorine, and ‘X’ represents anions, I^–^, Br^–^, Cl^–^, and HCOO^–^. (C) Formation energy (*E*_f_) of iodide vacancy (*V*_I_) in different surfaces. (D) Binding energy (*E*_b_) between the *V*_I_ in the FAI-terminated surface of Cs_0.05_FA_0.85_MA_0.1_PbI_2.9_Br_0.1_ (Cs, cesium; FA, formamidinium; MA, methylammonium) and various anions. (E) Structure of 2PACz and DBZ-2PACz. (F) Band edge positions extracted from UPS measurements. *E_Vac_* is vacuum level, *E_F_*is Fermi level, HOMO is the highest occupied molecular orbital, and VBM is the valence band maximum. (G) DFT-predicted π-π interaction between 4-F-PEAFa and 2PACz or DBZ-2PACz. The side view is shown here and the inset values denote the binding energy.

We proposed the construction of dual molecular bridges at perovskite heterointerfaces based on the following considerations: (1) The additive with anchoring groups could function as bridges, with tight bonding to perovskite film and stretching out to interact with the adjacent charge transporters [20,21]. (2) The tailored design of charge transport materials could enhance the interaction with the extended functional groups of the additive, thereby optimizing interfacial properties [22]. (3) The constructed molecular bridges could offer additional benefits, such as improved perovskite crystallization, defect passivation, and energy level alignment [19,20]. These concepts are illustrated in Fig. 1A. Therefore, we first start screening perovskite additives from 12 fluorinated organic salts (*n*-F-PEAX, *n* = 2, 3 and 4; X = I^–^, Br^–^, Cl^–^, and HCOO^–^, Fig. 1B). These additives were systematically assessed by density functional theory (DFT) calculations (Note S1) [23]. The fluorinated cations (2-F-PEA^+^, 3-F-PEA^+^, and 4-F-PEA^+^) possess a smaller size of NH_3_^+^ when compared to FA^+^, leading to stronger adsorption with the PbI_2_ subsurface layer and more stable *n*-F-PEAI-terminated surfaces of the perovskite (Fig. S1, Video S1). Figure 1C shows higher formation energies (*E*_f_) of iodide vacancies (*V*_I_) on *n*-F-PEAI-terminated surfaces (2.06–2.35 eV vs 1.90 eV for a pristine FAI-terminated surface), in which the maximum *E*_f_ suggests the fewest *V*_I_ on the 4-F-PEAI-terminated surface. It is noteworthy that the dipole moment of *n*-F-PEA increases with *n* and exhibits a strong linear relationship with its dimerization energy and the *V*_I_ formation energy on the *n*-F-PEAI-termination surface (Table S2 and Fig. S2). This phenomenon can be explained by the fact that a greater dipole moment leads to a more polarizable electron cloud, thereby strengthening van der Waals interactions and contributing to a more stable surface. Because the FAI-terminated surface is expected to be the dominant one in experiments [24], we further examine the passivation of iodide vacancies in the FAI-terminated surface by HCOO^–^ and different halide anions (Cl^–^, Br^–^, I^–^). The binding energies (*E*_b_) between the surface *V*_I_ and I^–^, Br^–^, Cl^–^, HCOO^–^ were determined to be –2.58, –2.84, –3.13, and –3.20 eV, respectively (Fig. 1D). Amongst them, HCOO^–^ exhibits the strongest binding with surface *V*_I_. Other defects in the minor PbI_2_-terminated surface [25], such as Pb_I_ antisites and FA interstitials can also be efficiently passivated by HCOO^–^ (Fig. S3). Therefore, the 4-F-PEACOOH (abbreviated as 4-F-PEAFa in the following sections) demonstrated an optimal combination of cation and anion in these fluorinated organic salts (evidenced in Figs S4 and S5). To link the perovskite layer and the carrier transport layers, the incorporation of phenyl units within the perovskite additive of 4-F-PEAFa and the selection of compatible carrier transport materials are imperative. The 4-F-PEA^+^ cations absorbed on perovskite surfaces have the capability to extend and establish a connection with the adjacent carrier transporters. Consequently, molecular bridges can be constructed. Molecular bridge I significantly facilitates electron transport (Fig. S6), due to the strong interaction between 4-F-PEA^+^ and C_60_ (Fig. S7) and the firm adsorption of 4-F-PEA^+^ to perovskite.

Then, we initiated the optimization process for the hole transport materials. We synthesized a derivative of the 2-(9H-carbazol-9-yl)ethyl]phosphonic acid (2PACz), namely [2-(7H-dibenzo[c, g]carbazol-7-yl)ethyl]phosphonic acid (DBZ-2PACz [26], Fig. 1E, Fig. S8) as a more adapted hole transporter for the construction of molecular bridge II. It is noted that these two molecules are considered due to the phenyl units that can form π-π interactions with the addition of 4-F-PEAFa and the group of phosphonic acids that can be fixed to the ITO surface. Herein, DBZ-2PACz has a deeper band edge (0.91 eV) as compared to 2PACz (0.78 eV) (Fig. 1F, Fig. S9). This reduced energy offset when in contact with the perovskite contributes to the enhanced *V*_OC_ and FF in our fabricated devices (Fig. S10). Furthermore, DFT results indicate a stronger interaction between 4-F-PEAFa and DBZ-2PACz (Δ*E*_π-π_ = –0.74 eV) than 2PACz (Δ*E*_π-π_ = –0.62 eV) (Fig. 1G, Fig. S11). Enabled by such a stronger π-π interaction, 4-F-PEAFa can well bridge the hole transporter and perovskite at the buried interface, which could lead to a significant reduction in both non-radiative recombination losses and carrier transport losses at this interface, as verified by the time-resolved photoluminescence (TRPL) measurements [27] (Fig. S12). These characteristics reaffirm the advantages and effectiveness of constructing molecular bridge II.

The existence of molecular bridges at perovskite interfaces can be further investigated by X-ray photoelectron spectroscopy (XPS) and X-ray diffraction (XRD). As shown in Fig. S13, the mixed thin films of 4-F-PEAFa/DBZ-2PACz and 4-F-PEAFa/C_60_ exhibit a stronger π→π* shakeup satellite compared to individual components in C1s XPS spectra. Additionally, when mixed with DBZ-2PACz, certain peak intensities of 4-F-PEAFa show a significant decrease in XRD patterns (Fig. S14), suggesting an interaction between 4-F-PEAFa and DBZ-2PACz. These findings confirmed the significant π-π interactions between 4-F-PEAFa and C_60_ or DBZ-2PACz transporters, leading to the formation of the molecular bridges. Furthermore, 4-F-PEAFa can affect the energy level of the perovskite (Figs S15 and S16), leading to different band positions at both buried and top interface. At the buried interface contacting DBZ-2PACz, the higher valence band maximum (VBM) indicated a facilitated hole transfer thanks to the reduced energy offset, consistent with Fig. 1F. At the top surface, 4-F-PEAFa induced a deeper VBM, which can act as a hole blocking effect [3]. These characteristics enable improved charge transport and reduced recombination losses at both perovskite heterointerfaces.

### Characterizations of perovskite films

**Figure 2.**
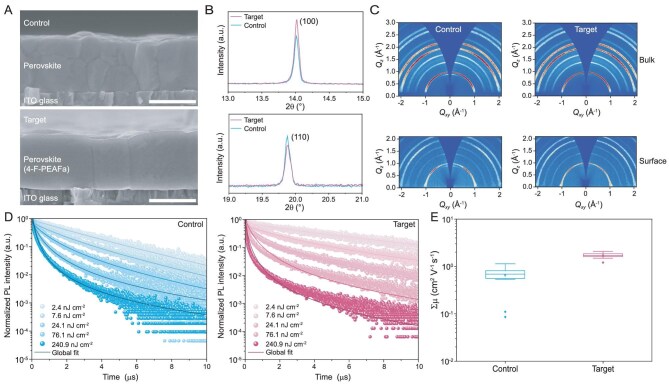
Characterization of perovskite films with or without adding 4-F-PEAFa. (A) Cross-sectional SEM images. The scale bars are 500 nm. (B) Enlarged XRD patterns. All films were deposited on the DBZ-2PACz-coated ITO glass. (C) 2D GIWAXS patterns for control and target films, measured at incidence angles *α_i_* = 0.5° for ‘surface’ measurements and = 2° for ‘bulk’ measurements, corresponding to attenuation lengths of 73 nm and 345 nm, respectively. (D) Fluence-dependent TRPL decays incident from the quartz side excited at 398 nm. Note that the perovskite films were deposited on the quartz. (E) Sum of charge carrier mobility of perovskite films obtained from the TPC measurements. Twelve data points from 2 samples at 6 different laser fluences were collected for each condition.

To confirm the functions of 4-F-PEAFa, we conducted a range of characterizations based on the as-prepared perovskite films (referred to as ‘control’) and those with the additive (referred to as ‘target’). Compared to the control film, the target film with a compact and uniform morphology has shown larger grain sizes (see the top-view scanning electron microscopy (SEM) in Fig. S17, Note S2). Cross-sectional SEM images (Fig. 2A) show a monolithic grain structure extending throughout the entire film (600–650 nm). XRD patterns of the target film exhibit a higher intensity along the [100] direction or (100) plane and an enhanced ratio of (100)/(110) (1.38 vs 0.84, obtained from the integrated peak intensity) (Fig. 2B, Fig. S18). Grazing-incidence wide-angle X-ray scattering (GIWAXS) measurements confirm that the target film has reduced (110) intensity and increased (100) intensity in the out-of-plane direction (Fig. 2C). Additionally, the target film exhibits a reduced monomolecular recombination rate constant (*k*_1_, from 0.9 × 10^5^ to 0.5 × 10^5^ s^−1^, Note S3) as determined by the TRPL measurements (Fig. 2D), and improved long-range charge carrier mobilities (sum-of-mobility of electron and hole, *∑μ*, from 0.65 to 1.71 cm^2^ V^−1^s^−1^, Note S4) as confirmed by transient photoconductivity (TPC) measurements (Fig. 2E). These gains in carrier dynamic characteristics could be attributed to the enhanced crystallinity of the target film as revealed above [28,29].

### Modification of 4-F-PEAFa on perovskite heterointerfaces

**Figure 3.**
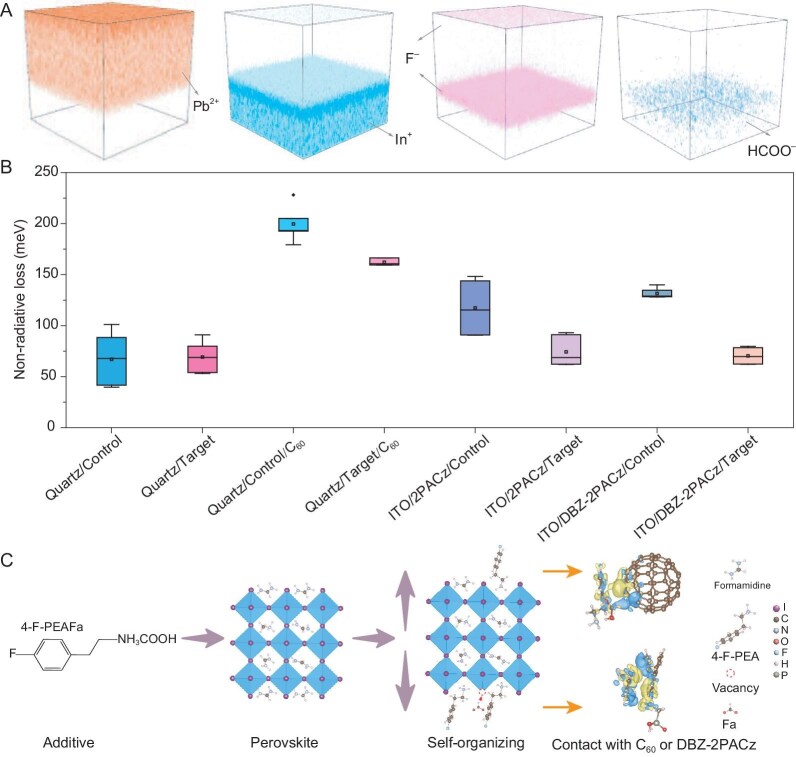
Self-organizing 4-F-PEAFa and its effect on perovskite with or without contacting charge transporters. (A) 3D depth profiles of ion distributions obtained from the ToF-SIMS measurements. (B) Non-radiative losses of perovskite films on different types of substrates or contacts. (C) Illustration of the self-organizing phenomenon of 4-F-PEAFa and the charge density difference. The red color circle (dash line) denotes the iodide vacancy and the Fa is prone to occupy this position. The 4-F-PEA occupies the FA at the dual sides of the perovskite. The blue regions represent electron accumulation, while the yellow regions indicate electron loss.

We further performed time-of-flight secondary-ion mass spectrometry (ToF-SIMS) to detect the distribution of 4-F-PEAFa in the target film as shown in Fig. 3A. The positive ions of Pb^2+^ and In^+^ denote the spatial location of perovskite and indium tin oxide (ITO) glass, respectively, whereas the negative ions of F^–^ and HCOO^–^ can be used to trace the 4-F-PEAFa. Interestingly, the F^–^ has a bimodal distribution, accumulating on both top (also confirmed in Fig. S19) and buried interfaces, whilst the HCOO^–^ is mainly located at the buried interface. We assume this phenomenon is caused by the species’ properties. A small amount residing near the top surface could be ascribed to the large size of 4-F-PEA^+^ [30], which cannot enter the perovskite structure and will thus stay at the surface during initial nucleation (see details in Fig. S20). The pseudohalide HCOO^–^ could alter the Pb coordination environment and thus the perovskite crystallization [21]. Hence, it is more likely to aggregate near the bottom given the top-down growth of the antisolvent-assisted spin-coated perovskite film [31]. To gain insight into this phenomenon, we calculated the dissociation energy for each organic salt component in the solution and found that 4-F-PEAFa is supposed to retard the perovskite crystallization by forming stable 4-F-PEAI and Pb(HCOO)_2_ with respect to FAI and PbI_2_ (Table S3). Therefore, 4-F-PEAFa shows spontaneous distribution or ‘self-organizing’ at both interfaces.

To understand the effects of such a self-organizing additive, we studied the carrier dynamics of the perovskite films under different conditions. When the perovskite is in contact with charge transporters, a reduction of photoluminescence quantum yield (PLQY) is observed (Fig. S21). This is consistent with interfacial recombination as previously reported [32]. Encouragingly, with the incorporation of 4-F-PEAFa in perovskite film, the PLQY at both interfaces are substantially recovered. The calculated quasi-Fermi level splitting (QFLS, Figs S22 and S23, Note S5) and non-radiative losses (Fig. 3B, Note S6) confirm a reduction of non-radiative recombination at both heterointerfaces. Hence, the 4-F-PEAFa not only modifies the perovskite bulk but also plays a significant function at perovskite heterointerfaces. These effects are closely related to the spatial regulation of 4-F-PEAFa. Specifically, at the buried interface, the accumulation of 4-F-PEA^+^ and HCOO^–^ enable the suppression of perovskite defects as we discussed earlier in the DFT calculations, thereby effectively reducing interfacial losses. At the top interface, the large organic 4-F-PEA^+^ does not form a 2D phase, with no detectable scattering ring in 2D GIWAXS patterns (Fig. 2C) or peaks in the 1D integrated data (Fig. S24). Nonetheless, the π-conjugation structure of 4-F-PEA^+^ leads to good stacking with the C_60_ (Fig. S7), facilitating electron transport between the perovskite layer and the top electrodes (Fig. S6). Therefore, the terminal groups of 4-F-PEA^+^ provide favourable connection with both the C_60_ and DBZ-2PACz to achieve dual molecular bridges. This will lead to the development of an efficient integral carrier transport pathway throughout the entire device. Further calculated charge density difference indicated the charge transfer at both perovskite heterointerfaces (Fig. 3C), which could be beneficial for the performance of the device.

### Photovoltaic performance

**Figure 4.**
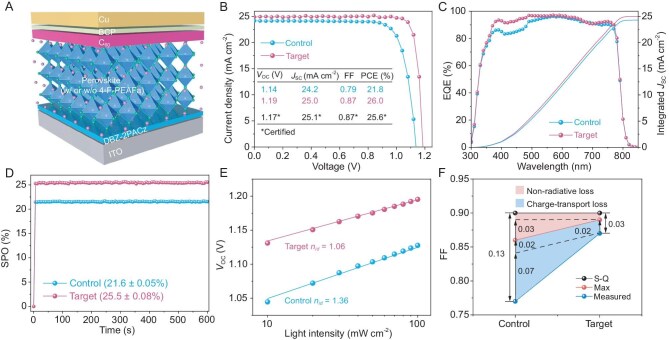
Characterizations of unencapsulated IPSCs with or without adding 4-F-PEAFa. (A) Schematic configuration of the device. (B) Champion current density-voltage (*J-V*) curves under AM 1.5 G simulated solar illumination. (C) EQE spectra. (D) Stabilized power output (SPO) determined at *V*_MPP_. For SPO, the devices were measured under the AAA solar simulator (Xenon lamp, without UV filter). The *V*_MPP_ of the control and target is 0.95 and 1.06 V, respectively. (E) Light intensity dependency of *V*_OC_. The ideality factor (*n*_id_) is obtained from the linear fit. (F) The analysis of the FF losses, including the Shockley–Queisser (S-Q), maximum (max) and measured FF. All calculation details can be found in the Supplementary materials. The blue area denotes the charge-transport loss and the pink area denotes the non-radiative loss. The dashed lines help to indicate the reduced FF losses.

To verify the effects of molecular bridges, we fabricated >700 devices with a structure of glass/ITO/DBZ-2PACz/perovskite/C_60_/bathocuproine (BCP)/Cu (Fig. 4A, Fig. S25). The presence of DBZ-2PACz in IPSCs as the hole transporter was detected in the depth profile (Fig. S26). Statistical data (Fig. S27) confirmed a remarkable performance improvement between the control (without 4-F-PEAFa) and target devices (with 4-F-PEAFa) and excellent repeatability, in which the champion efficiency increased from 21.8% to 26.0% and the champion FF increased from 0.83 to 0.87 (Fig. 4B). Compared to the control devices, our target devices exhibit improved homogeneity, as observed in the statistical data. Such high uniformity is crucial for the industrialization of perovskite photovoltaics, directly impacting large-scale reproducibility and commercial viability. The significant enhancement in homogeneity can be attributed to the incorporation of dual molecular bridges. Our modifications to perovskite adjacent interfaces resulted in more uniform carrier extraction and reduced local defects, leading to lower device-to-device variability, which is critical for large-area scalability (Fig. S28).

To verify the photovoltaic parameters measured in our lab, we sent five high-performing devices to an accredited laboratory for certification. The champion performance yields a *V_OC_*of 1.17 V, a *J*_SC_ of 25.1 mA cm^−2^, an FF of 0.87 and a PCE of 25.6% in the reverse scan; a *V_OC_*of 1.17 V, a *J*_SC_ of 25.1 mA cm^−2^, an FF of 0.86 and a PCE of 25.2% in the forward scan (Fig. S29). These devices reached an average PCE of 25.1% (25.1% ± 0.35%, obtained from both forward and reverse scans in Table S4). To the best of our knowledge, the certified PCE of 25.6% and FF of 0.87 (96% of its Shockley–Queisser limit for a PSC with a 1.57 eV bandgap) achieved in this study are amongst the highest photovoltaic parameters reported to date (see more details in Table S1 and Fig. 4B). Additionally, such certified high FF (the maximum is 0.88, with an average of 0.87 ± 0.01 in Table S4) and the product of *V_OC_* × FF (88% of the Shockley–Queisser limit) indicate an extremely efficient collection of photo-generated carriers from the perovskite into the charge transport layers [14].

We calculated the integrated *J*_SC_ from external quantum efficiency (EQE) spectra where the control and target device show 24.3 mA cm^−2^ and 25.0 mA cm^−2^, respectively (Fig. 4C), with a negligible mismatch between the *J-V* and EQE measurements. Figure 4D shows the steady-state power output (SPO) at the maximum power point voltage (*V*_MPP_). The steady-state PCEs (21.6% ± 0.05% for the control and 25.5% ± 0.08% for the target) closely match the *J-V* scan-determined PCEs. Compared to the control, the target device shows a reduced voltage deficit (defined by *E*_g_*/q—V*_OC_, where *E*_g_ is the bandgap, 1.57 eV from Fig. S30) from 0.43 to 0.38 V. More significantly, light-intensity dependent *V*_OC_ measurements exhibit a suppressed ideality factor (*n*_id_ = 1.06 for the target device, Fig. 4E and Fig. S31), which is the lowest value among the high-performance PSCs (Table S1). Both characteristics demonstrate high perovskite quality and minimal interfacial losses [33].

It is known that the FF can be affected by the sweep direction due to hysteresis in *J-V* curves. Here, both statistical lab-measured (Fig. S27) and certified (Fig. S29, Table S4) data show the stable variation of the FF either from forward or reverse scans, making them ideal candidates for quantitative analyses. To understand the FF improvement, *n*_id_ values and the corresponding *J-V* data were further exploited. The maximum FFs (without considering resistance) of the control and target devices are 0.86 and 0.89 (Fig. 4F, Note S7), approaching the Shockley–Queisser limit value of 0.903 at a 1.57 eV band gap. After introducing the equivalent series (control vs target, 3.57 vs 1.41 Ohm·cm^2^) and shunt resistances (6840 vs 10 602 Ohm·cm^2^), the calculated FFs (0.79 vs 0.86) are in good agreement with the measured FFs (0.77 vs 0.87) for the control and target devices. In comparison with the reduced non-radiative losses of 0.03, the charge-transport losses are significantly inhibited by 0.07 in the target device. This phenomenon could be attributed to the properties of the top-down carrier viaduct, which relies on two molecular bridges positioned on both sides of the perovskite layer. The total FF loss significantly decreased from 0.13 to 0.03, demonstrating the effectiveness of the approach in this study.

### Long-term stability

**Figure 5.**
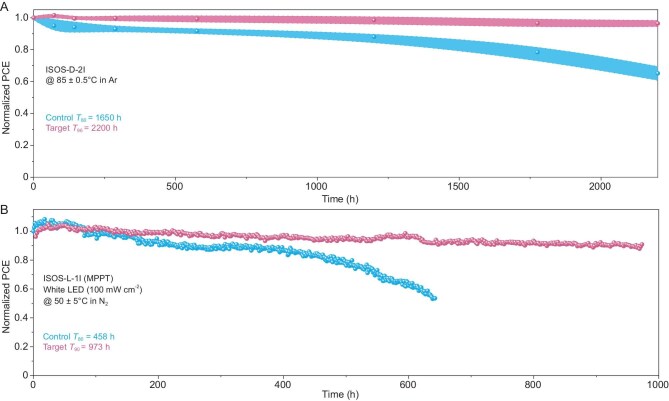
Long-term stability under (A) thermal or (B) maximum power point tracking. Note that five devices for each group were measured for thermal stability. The width of the shaded regions represents the standard deviation of the PCEs.

We further investigated the long-term performance stability of our devices without any encapsulation under three conditions based on the International Summit on Organic Photovoltaic Stability (ISOS) protocols [34]. In ISOS-D-1 testing (Fig. S32), the *T*_85_ (lifetime at 85% of the initial efficiency) of devices is enhanced from 271 to 1128 h with 4-F-PEAFa modification. This may be related to improved robustness to moisture, with contact angle measurements confirming a more hydrophobic surface of the 4-F-PEAFa–containing perovskite (Fig. S33). Furthermore, over 2200-h ISOS-D-2I testing (Fig. 5A), the target devices show better thermal resistance with a *T*_96_ (lifetime at 96% of the initial efficiency) of 2200 h. In contrast, the control devices only retain a *T*_80_ (lifetime at 80% of the initial efficiency) of 1650 h. Determined at maximum power point (Fig. 5B), the ISOS-L-2I tracking shows a *T*_80_ of 458 h for the control device and a *T*_90_ of 973 h for the target device. This enhanced operational stability could be ascribed to the passivation effect of the molecular bridges at adjacent interfaces, which are beneficial for inhibiting ion migration under heat or light stressors [5]. Taken together, the results demonstrate that our rationally designed molecular bridges at perovskite adjacent heterointerfaces simultaneously improved the photovoltaic performance and stability of IPSCs.

## CONCLUSIONS

In this work, we successfully construct dual bridges at perovskite heterointerfaces through the addition of 4-F-PEAFa, which can be firmly absorbed on the perovskite surface and interacts with the electron transporter (C_60_) at the top interface and synergistically links with a customized hole transporter (DBZ-2PACz) at the bottom interface. Such a molecular design approach contributes to reduction of non-radiative recombination and charge-transport losses at perovskite heterointerfaces through the formation of an efficient carrier transport pathway in the complete devices, resulting in inverted perovskite solar cells with a certified efficiency of 25.6% and an exceptional fill factor of 0.87, along with excellent thermal and operational stability. Our study highlights the significance of the integral design of molecular bridges and provides useful guidance in the commercialization of these promising photovoltaic devices.

## Supplementary Material

nwaf211_Supplemental_File

## Data Availability

The datasets generated during and/or analyzed during the current study are available from the corresponding author on reasonable request.
