# Supplementary material for: Dual molecular bridges at perovskite heterointerfaces for efficient inverted solar cells
Source: Natl Sci Rev. 2025 May 22;12(7):nwaf211. doi: 10.1093/nsr/nwaf211 (PMC12203910; doi:10.1093/nsr/nwaf211)
Supplement: nwaf211_Supplemental_File [file nwaf211_supplemental_file.pdf]

Supplemental Information

Dual molecular bridges at perovskite heterointerfaces for efficient inverted solar cells

Qing Lian[1,†], Lina Wang[1,†], Guoliang Wang[1,4,†], Guojun Mi[1,†], Bowei Li[5,*], Joel A. Smith[3], Pietro Caprioglio[3], Manuel Kober-Czerny[3], Deng Wang[1], Qiming Yin[1], Jiong Yang[6], Sibo Li[7], Xiao Liang[8], Shaokuan Gong[7], Dongyang Li[1,9], Hanlin Hu[8], Xihan Chen[7], Xugang Guo[1], Longbin Qiu[7], Baomin Xu[1], Gang Li[9], Anita W. Y. Ho-Baillie[4], Wei Zhang[2,10,*,‡], Guangfu Luo[1,*], Henry J. Snaith[3,*] and Chun Cheng[1,7,11*]

[1]Department of Materials Science and Engineering, Southern University of Science and Technology, Shenzhen 518055, China;
[2]Advanced Technology Institute, Department of Electrical and Electronic Engineering, University of Surrey, Guildford GU2 7XH, UK;
[3]Clarendon Laboratory, Department of Physics, University of Oxford, Oxford OX1 3PU, UK;
[4]School of Physics and The University of Sydney Nano Institute, The University of Sydney, Sydney, NSW 2006, Australia;
[5]Future Photovoltaic Research Center, Global Institute of Future Technology, Shanghai Jiao Tong University, Shanghai 200240, China;
[6]Department of Chemistry, Southern University of Science and Technology, Shenzhen 518055, China;
[7]SUSTech Energy Institute for Carbon Neutrality, Department of Mechanical and Energy Engineering, Southern University of Science and Technology, Shenzhen 518055, China;
[8]Hoffman Institute of Advanced Materials, Shenzhen Polytechnic, Shenzhen 518055, China;
[9]Department of Electronic and Information Engineering, Research Institute for Smart Energy, The Hong Kong Polytechnic University, Hong Kong 999077, China;
[10]State Centre for International Cooperation on Designer Low-carbon & Environmental Materials (CDLCEM), School of Materials Science and Engineering, Zhengzhou University, Zhengzhou 450001, China
[11]Guangdong Provincial Key Laboratory of Energy Materials for Electric Power, Southern University of Science and Technology, Shenzhen 518055, China.
***Corresponding authors**. E-mails: bowei23@sjtu.edu.cn; wz0003@surrey.ac.uk; luogf@sustech.edu.cn; henry.snaith@physics.ox.ac.uk; chengc@sustech.edu.cn
†Equally contributed to this work.
‡ Lead contact

The PDF file includes:
Supplementary Text
Figure S1 to S33
Table S1 to S4
Note S1 to S7
References

Methods

Materials

2-(4-fluorophenyl)ethylamine (4-F-PEA, >98%), 2-(3-fluorophenyl)ethylamine (3-F-PEA, >98%), 2-(2-fluorophenyl)ethylamine (2-F-PEA, >98%), formic acid (>98%), [2-(9H-Carbazol-9-yl)ethyl]phosphonic acid, 7*H*-dibenzo[*c,g*]carbazole were procured from TCI, P. R. China. C_{60} (99%) was purchased from Nano-C Inc. Formamidinium iodide (FAI, 99.9%), methylammonium chloride (MACl, 99.9%), methylammonium bromide (MABr, 99.9%), lead iodide (PbI_2, 99.999%) were purchased from Advanced Election Technology Co., Ltd. All *n*-F-PEAX (*n* = 2, 3, 4 and X = I, Br, Cl) were purchased from Xi'an P-OLED. Piperazinium diiodide (PDI) was synthesized according to the previous report.[1] Bathocuproine (BCP, 99.99%), lead chloride ($PbCl_2$, 99.999% trace metals basis), N,N-dimethylformamide (DMF, anhydrous, 99.8%), dimethyl sulfoxide (DMSO, anhydrous, 99.9%), 1-Methyl-2-pyrrolidinone (anhydrous, 99.5%), methanol (HPLC, 99.9%), ethanol (anhydrous), 2-propanol (anhydrous, 99.5%) and ethyl acetate (anhydrous, 99.8%) were obtained from Sigma-Aldrich (P. R. China). All solvents were deoxygenated in a N_2-filled glovebox for a minimum of 2 h to prevent the effect of oxygen levels during the spin-coating process.

n-F-PEAFa

2-(*n*-fluorophenyl)ethylamine formate (*n*-F-PEAFa) was synthesized by drop by drop adding 1 M formic acid into 1 M 2-(*n*-fluorophenyl)ethylamine solution in ethanol with vigorous stirring. After stirring for 270 min in the ice bath, diethyl ether was added to precipitate a white powder. The resulting powder was then dissolved in ethanol and precipitated using diethyl ether. This procedure was repeated 4–6 times to improve purity, and the white powder was recrystallized from ethanol. The resulting powders were dried at 60 °C for 24 h under the vacuum.

Perovskite solution

$Cs_{0.05}FA_{0.85}MA_{0.1}PbI_{2.91}Br_{0.09}$ was prepared according to our previous procedure.[2] Briefly, PbI_2 (750 mg), FAI (225 mg), MABr (16 mg), MACl (12 mg) and CsI (20 mg) were mixed in a 4 mL vial at solvent free nitrogen filled glovebox and dissolved in 1050 µL DMF:DMSO (5:1, v/v) at non-solvent controlled nitrogen glovebox. Then, the perovskite precursor was vigorously stirred until dissolved (normally in 10 min) and immediately filtered with a 0.22 µm PTFE filter. For additive-containing perovskite, 10 µL of *n*-F-PEAFa (1M, dissolved in DMF as a stock solution) was added into the precursor before stirring.

Device fabrication

Hole-extraction layer. Patterned ITO glass substrates were first cleaned by soap, acetone, absolute ethanol and 2-propanol in the ultrasonic bath until cleaned, and then treated with UV-ozone for 20 min before use. 40 µL of 2-(9H-carbazol-9-yl)ethyl]phosphonic acid (2PACz) or [2-(7H-dibenzo[c,g]carbazol-7-yl)ethyl]phosphonic acid (DBZ-2PACz) solution (dissolved in anhydrous methanol at 1 mmol mL^{-1}) was spin-coated onto the ITO substrates at 4000 rpm for 5 s and then transferred to a hot plate (IKA RCT digital) at 20 °C and raised to 100 °C for 10 min.

After the allotted time, the substrates were then quickly removed from the hot plate. We noted that the wettability of the film will deteriorate with the increase of annealing time. Before perovskite deposition, the substrates were washed with ethanol.

Perovskite layer. 40 μL of perovskite solution ($Cs_{0.05}FA_{0.85}MA_{0.1}PbI_{2.91}Br_{0.09}$) was spread on the 2PACz- or DBZ-2PACz-coated substrate substrate and spin-coated at 1000 rpm for 10 s and 4000 rpm for 30 s. Then, 150 μL of ethyl acetate was dripped in the last 5–6 s. The perovskite film was then annealed on a hot plate at 65 °C for 5 min with an inverted petri dish and 120 °C for 20 min. For high-performance devices (see details in Figure S22, S23, Table S4), a saturated PDI solution (2 mg in 4 mL IPA, dissolved at 100 °C) was dynamically spin-coated on the perovskite surface at 6000 rpm for 5 s and annealed at 105 °C for 5 min.

Electron-extraction layer and metal electrodes. C_{60} (40 nm), BCP (9 nm) and Cu (120 nm) were thermally evaporated under a high vacuum (< 2×10^{-4} Pa). The active area is 0.136 cm^2. For high-performance devices, an anti-reflection layer (MgF_2, 105 nm) is evaporated on the glass side. All the fabrication process was conducted in the cleanroom (16–19 °C). For the glovebox, it is crucial to maintain an oxygen level of less than 0.1 ppm and a temperature of less than 19 °C. We noted reduced efficiency when the oxygen and temperature are over the above values.

Characterization of the solar cells

The solar cells without encapsulation were measured in a N_2-filled glovebox. All *J-V* curves were measured using an Enlitech SS-X50 solar simulator with a 300 W Xenon lamp (Class AAA). The light intensity was calibrated to AM 1.5G (mismatch factor between 0.994 and 1) using a Si reference cell (Newport, Model: 91150V, SN: 1194) before measurements. All devices were measured in reverse (1.3 → −0.1 V, step 0.01 V, delay time 10 ms) and forward scan (−0.1 → 1.3 V, step 0.01 V, delay time 10 ms). To ensure accuracy, a mask with an aperture area of 0.084 cm^2 was used during the measurement. The stabilized power output (SPO) was measured at V_{MPP} under the same solar simulator in the glovebox. External quantum efficiency (EQE) measurements were performed in ambient air using QE-R EQE system (Enlitech) with a step size of 10 nm.

Stability test

The maximum power point tracking (MPPT) was conducted on a commercial system (91PVKSolar Co. Ltd, China). The devices were stored in a N_2-filled chamber at 50 ± 5 °C controlled by a hotplate. The illumination source is a white LED (equivalent to 1 Sun intensity) and the MPP is determined by the perturb and observe method. The thermal stability was performed on the hotplate at 85 ± 0.5 °C in a Ar-filled glovebox (O_2 < 0.1 ppm, H_2O < 0.1 ppm) under dark storage. The shelf-life stability was conducted in a humidity-controlled box (25 ± 5% RH) without light control.

Film characterization

Scanning electron microscope (SEM) images were conducted on Hitachi SU8230 with an accelerating voltage of 5 kV to obtain the surface and cross-sectional morphologies.

X-ray diffraction (XRD) patterns were obtained using a Rigaku SmartLab diffractometer with Cu Kα (λ = 1.5418 Å) radiation.

Time-resolved photoluminescence (TRPL) spectra were collected on a FluoTime 300 (PicoQuant GmbH) with a TimeHarp 260 as a time-correlated single photon counting setup (TCSPC) and a pulsed laser diode with an excitation wavelength of 398 nm (LHD-P-C-405, PicoQuant GmbH). The pile-up rate is kept below 5% for each measurement. All samples were measured with a 50 KHz repetition rate at a Fluence of ~ 24 nJ cm^{-2}. For the plots, the median number of counts before the laser absorption was subtracted from the data as background.

The long-range mobility was calculated from transient photo-conductivity (TPC) measurements. For this, interdigitated gold electrodes spaced 300 μm apart were evaporated on top of the neat perovskite samples on quartz. The samples were illuminated from the quartz side by a pulsed laser at 550 nm (10 Hz repetition rate) with Fluences from 61 to 19 μJ cm^{-2}. The mobility was then estimated from the photoconductivity at $t = 0$, using the excitation density as calculated from a film thickness of 550 nm and an absorptance of 75%. Further, the mobilities estimated at higher fluences are corrected, to include the effects of early-time recombination, as described in detail in a recent study.[3]

Photoluminescence quantum yield (PLQY). The absolute photoluminescent measurements were measured by exciting the sample with a CW laser source of 532 nm (ThorLabs DJ532-10) at 1 sun intensity equivalent inside an integrating sphere. All spectra are illuminated for the same amount of time of 5 s to improve consistency. The actual photoluminescence spectra are recorded through a Maya2000 Pro spectrometer by using Ocean View software.

X-ray photoelectron spectroscopy (XPS) and ultraviolet photoelectron spectroscopy (UPS) measurements were conducted using ESCALAB 250Xi (Thermo Fisher Scientific). A monochromatic aluminum (K_α) X-ray source provides photons with the energy of 1486.7 eV for XPS, and a standard helium-discharge lamp with He Iα photons at 21.22 V for UPS. The total energy resolution of XPS and UPS were 0.30 eV and 50 meV, respectively. The XPS data were analyzed and calculated using the Avantage software; The work function was extracted from the edge of the secondary electron cut-off of the UPS spectra by applying a bias voltage of –7 V to the sample. For UPS measurement of the buried perovskite, we detached the as-crystallized perovskite film from the substrate, following a previously reported method [4]. Briefly, we applied a UV-curable glue on top of the device, cured it with a cover glass, and then peeled off the film using a glass plier.

Time-of-flight secondary ion mass spectroscopy (ToF-SIMS) was conducted to evaluate the ion distributions in perovskite film. The films were tested using a ToF-SIMS instrument (ION-ToF GmbH, Germany) with a Bi^+ primary beam (30 keV and 1 pA) and a Cs^+ sputter beam (0.5 keV and 40 nA). The sputter size was 350 μm × 350 μm, and the analysis was 100 μm × 100 μm.

Grazing-incidence wide-angle X-ray scattering (GIWAXS) data were acquired with a Rigaku SmartLab diffractometer. A 3 kW Cu X-ray source (λ = 1.54 Å) was operated

at 40 kV, 45 mA with parallel beam optics configuration together with 1° in plane parallel slit collimator and 0.3° long collimator attachments to minimize the X-ray spot size (confirmed by measuring the direct beam). X-rays were incident on thin film perovskite samples prepared on quartz glass, which were mounted on a 2D-XRD attachment head, incorporating a knife-edge to prevent air scatter and an aligned beam stop for the direct beam. Scattering was collected with a HyPix-3000 hybrid pixel-array 2D detector with a sample-to-detector distance of 65 mm. Exposed sample widths were reduced to ~3 mm to minimize sample footprint broadening. Data was collected at grazing incidence angles (α_i) of α_i = 0.5° for 'surface' measurements and α_i = 2° for 'bulk' measurements, corresponding to attenuation lengths of 73 nm and 345 nm, respectively. For each measurement, the detector goniometer arm was rotated through 2θ angles from 0° to 40° in 1° steps for the bulk measurements and 16° to 17° in 0.1° steps for the surface, with 300 s acquisition at each position. Detector images were then remapped into Q-space and combined together using scripts based on the PyFAI and Pygix libraries, which were also used for 1D azimuthal profiles.[5] Simulated powder XRD patterns were calculated VESTA using previously reported structural data.[6],[7]

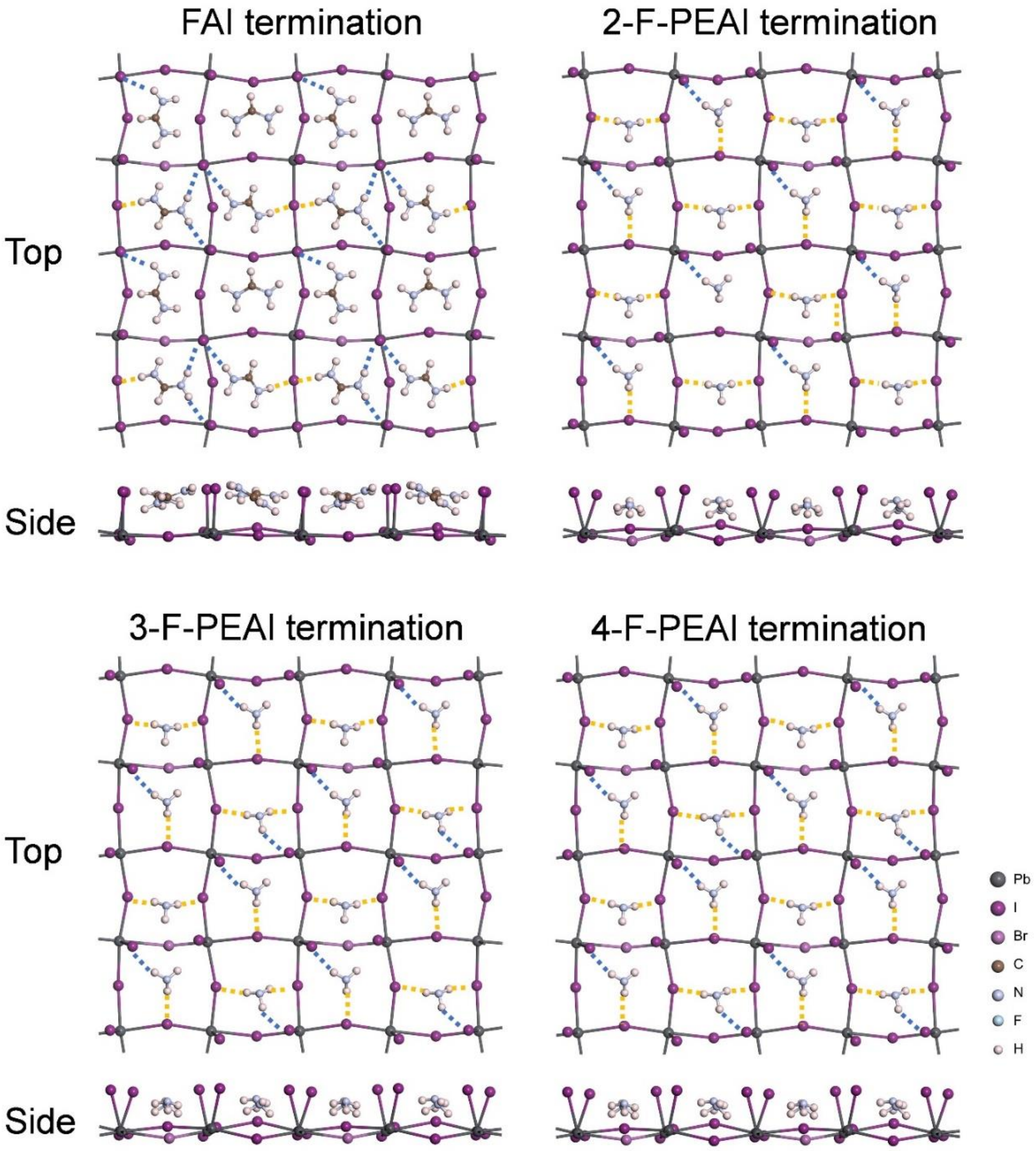

Figure S1. Local structures in the FAI- and *n*-F-PEAI-terminated surfaces of $Cs_{0.05}FA_{0.85}MA_{0.1}PbI_{2.90}Br_{0.10}$. For easy visualization, only the top two layers of each surface are illustrated, so does the group NH_3^+ of each *n*-F-PEA$^+$. Hydrogen bonds between H and I in the top and second layers are shown in blue and yellow dashed lines, respectively. The close distances between group NH_3^+ of *n*-F-PEA$^+$ and the PbI_2 subsurface layer lead to more hydrogen bonds with the PbI_2 layer than that of the corresponding FA$^+$. This phenomenon is consistent with the higher formation energy of V_I (Figure 1C) in the *n*-F-PEAI-terminaed surface than those of the FAI-terminated surface.

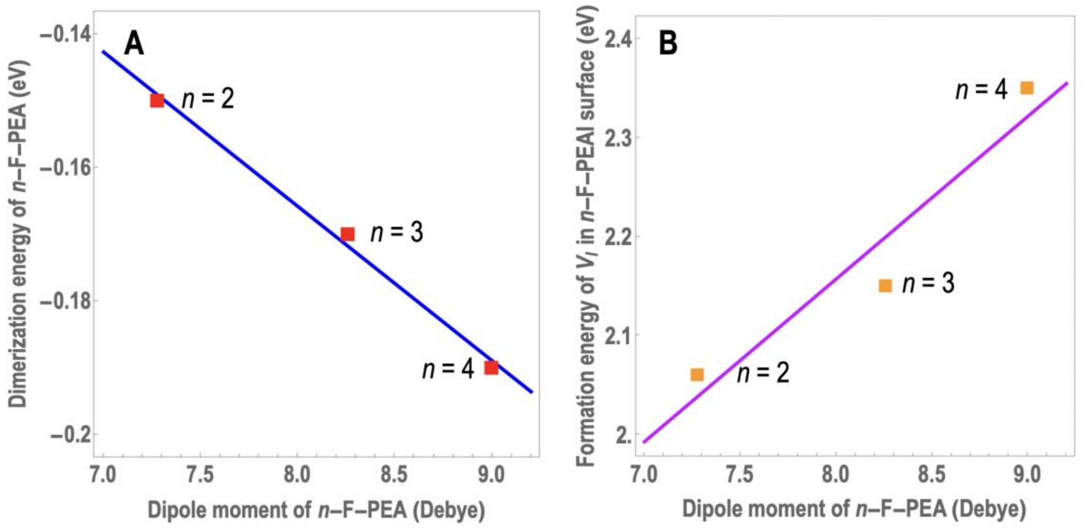

Figure S2. (A) Correlation between the dipole moment of *n*-F-PEA and the dimerization energy of *n*-F-PEA; (B) Correlation between the dipole moment of *n*-F-PEA and the formation energy of V_I in *n*-F-PEAI-termination surfaces.

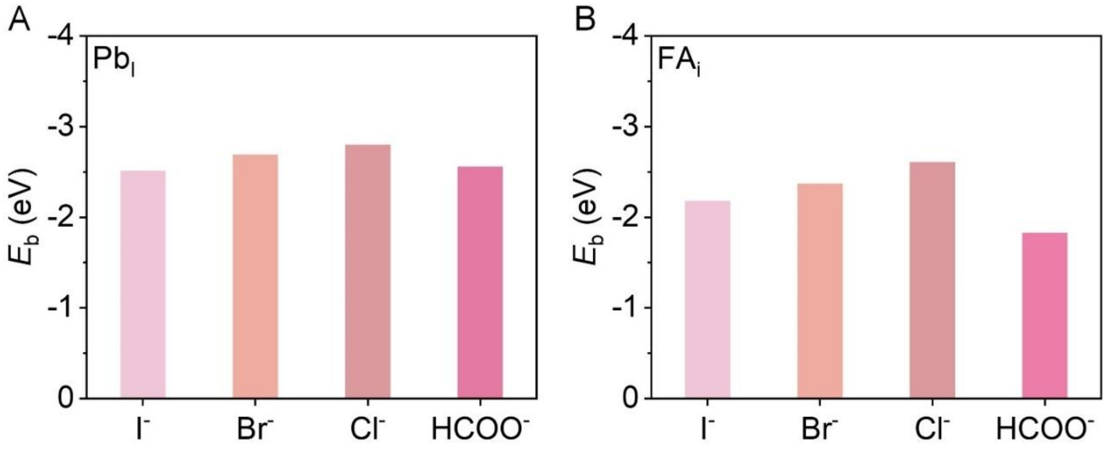

Figure S3. DFT-predicted binding energy between different anions and (A) antisite defect (Pb_I), or (B) FA interstitial defect (FA_i) in the less stable PbI_2-terminated surface.[8] These results show that all the anions can strongly bind with Pb_I and FA_i.

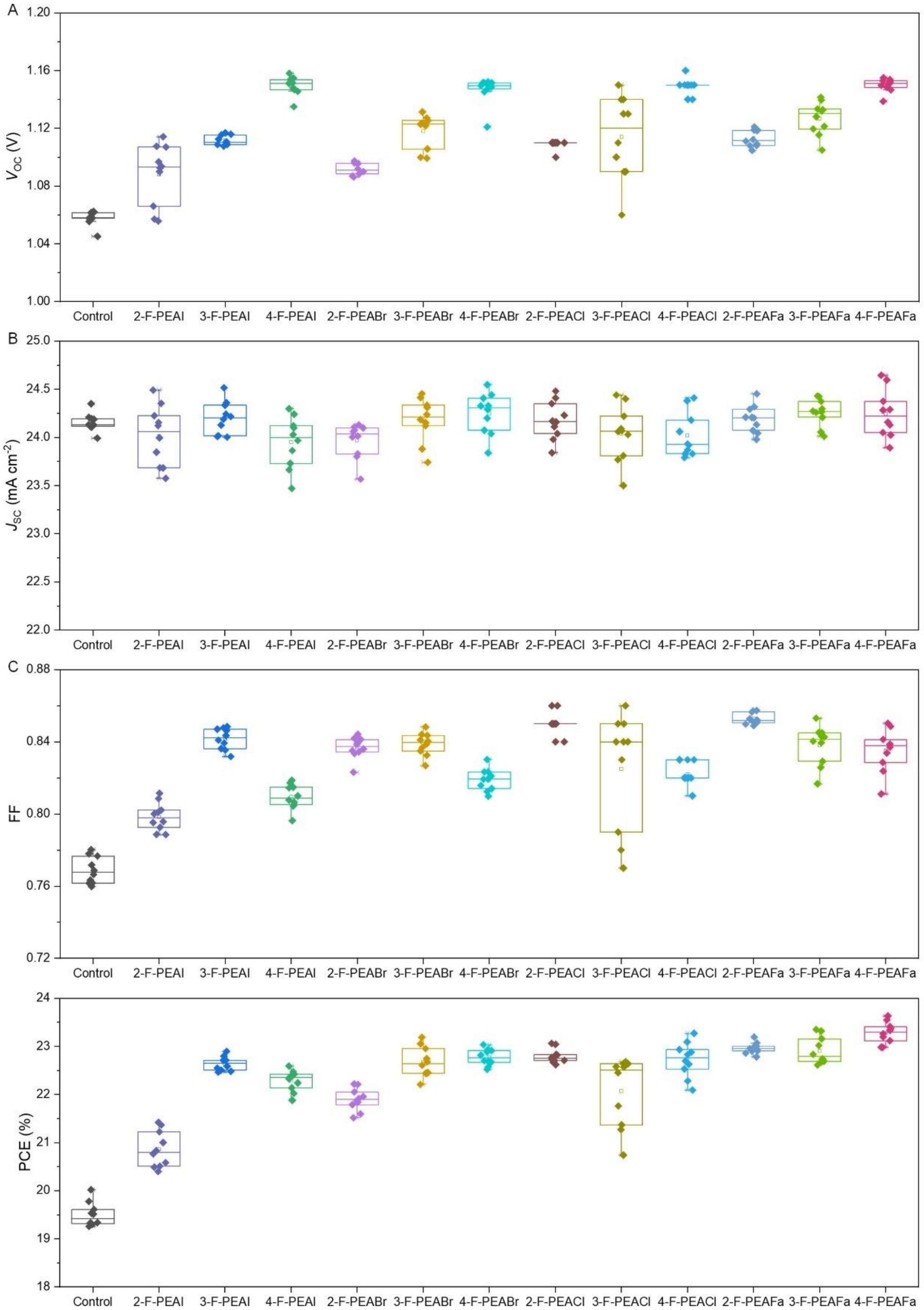

Figure S4. Statistical distributions of photovoltaic parameters (V_{OC}, J_{SC}, FF and PCE) based on the different *n*-F-PEAX (*n* = 2, 3, 4; X = I^{-}, Br^{-}, Cl^{-}, and $HCOO^{-}$). Note that 10 devices were measured for each condition with a structure of glass/ITO/2PACz/perovskite/C_{60}/BCP/Cu. 1 mol% *n*-F-PEAX was added to the perovskite solution with a composition of $Cs_{0.05}FA_{0.85}MA_{0.1}PbI_{2.91}Br_{0.09}$.

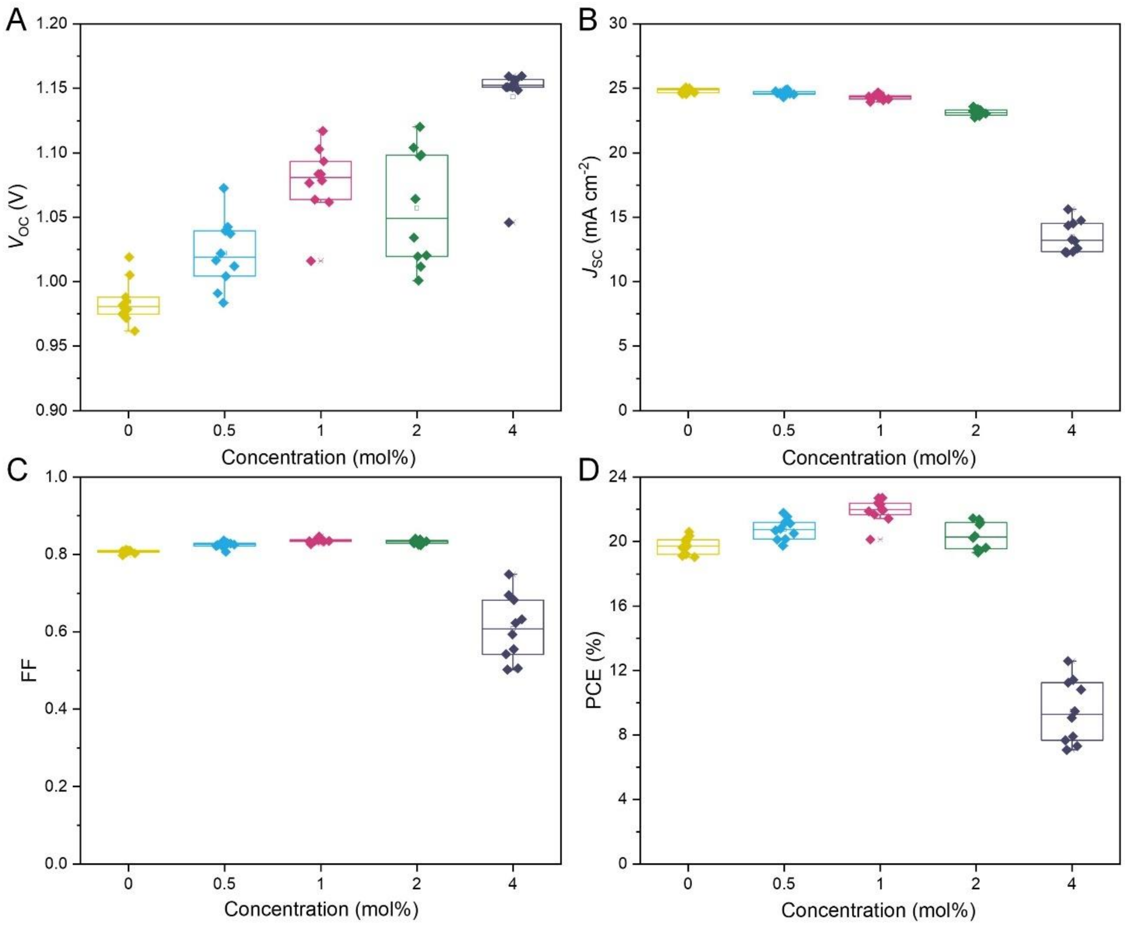

Figure S5. Statistical distributions of photovoltaic parameters (V_{OC}, J_{SC}, FF and PCE) based on the different concentration of 4-F-PEAFa. Note that 10 devices were measured for each condition with a structure of glass/ITO/2PACz/perovskite/C_{60}/BCP/Cu.

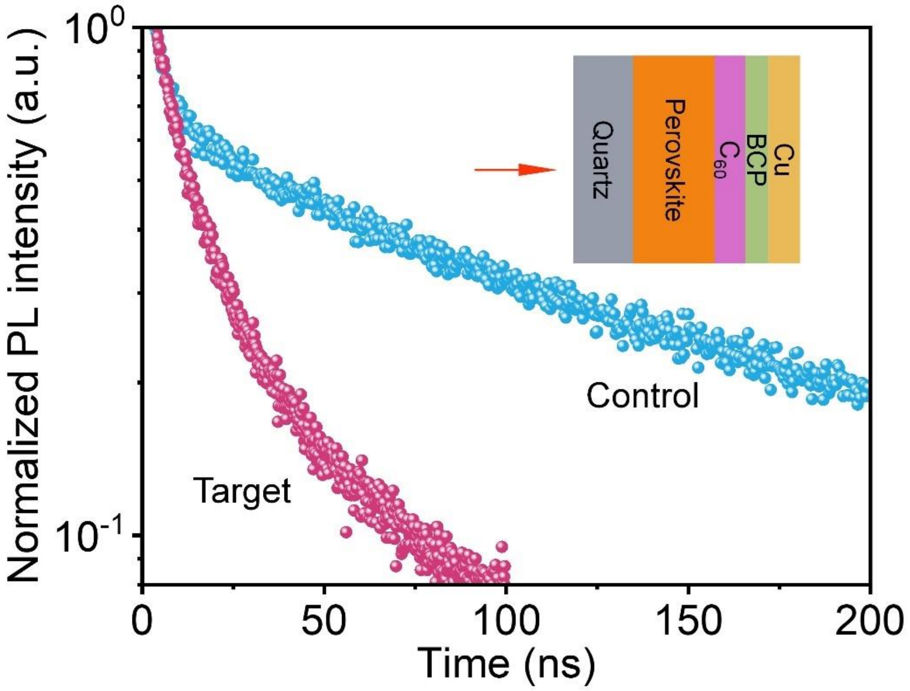

Figure S6. Time-resolved photoluminescence (TRPL) decay of half device. Note that the perovskite is prepared with (target) or without (control) 4-F-PEAFa.

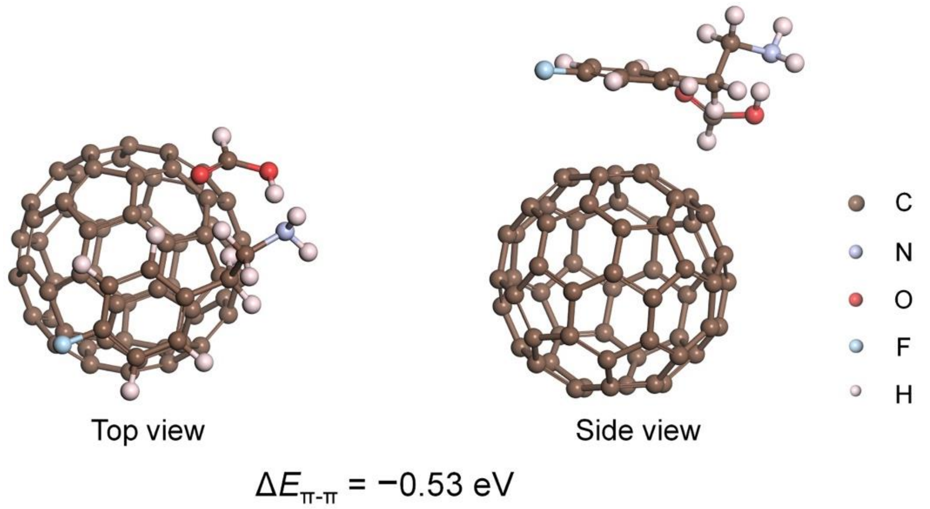

Figure S7. DFT-optimized structure of 4-F-PEAFa adsorbed on C_{60}. A binding energy of –0.53 eV suggests a relatively strong π-π interaction between 4-F-PEAFa and C_{60}.

DBZ-2PACz was synthesized similarly to the previous report:[9]

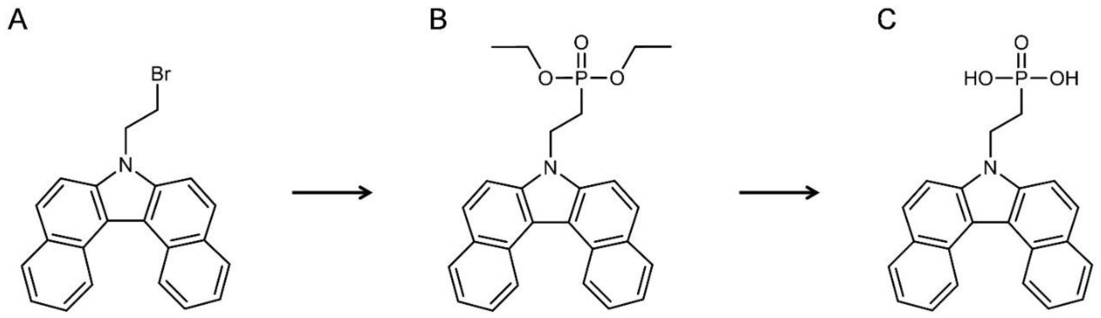

Figure S8. Synthesis scheme of DBZ-2PACz.

7-(2-bromoethyl)-7H-dibenzo[c,g]carbazole (Figure S8A): 7*H*-dibenzo[*c*,*g*]carbazole (10 mmol) was dissolved in 1,2-dibromoethane (0.8 mol), tetrabutylammonium bromide (1.5 mmol) and KOH solution (50 wt.% in water, 50 mmol) were added subsequently. The reaction was stirred at 60 °C overnight (~12 h). After completion of the reaction, extraction was done with dichloromethane. The organic layer was dried over anhydrous Na_2SO_4 and the solvent was distilled off under reduced pressure. The crude product was purified by column chromatography (*n*-hexane: ethyl acetate 20:1 *v:v*) to give 1.46 g of a colorless solid.

^{1}HNMR (400 MHz, $CDCl_3$): δ 9.23 (d, *J* = 8.5 Hz, 2H), 8.08 (dd, *J* = 8.1, 1.4 Hz, 2H), 7.96 (d, *J* =8.8 Hz, 2H), 7.73 (dd, *J* =9.1, 7.8 Hz, 4H), 7.57 (ddd, *J* =7.9, 6.8, 1.0 Hz, 2H), 4.94 (t, *J* =7.5 Hz, 2H), 3.75 (t, *J* =7.5 Hz, 2H).

^{13}C NMR (101 MHz, $CDCl_3$): 136.7, 129.9, 129.2, 129.1, 127.0, 125.6, 125.2, 123.5, 117.7, 110.3, 44.7, 28.5.

Anal. calcd for $C_{22}H_{16}BrN$, %: C 70.60, H 4.31, N 3.74, found, %: C 70.41, H 4.57, N 3.73.

Diethyl (2-(7H-dibenzo[c,g]carbazol-7-yl)ethyl)phosphonate (Figure S8B): 7-(2-bromoethyl)-7*H*-dibenzo[*c*,*g*]carbazole (3 mmol) was dissolved in triethyl phosphite (80 mmol) and the reaction mixture was heated at reflux overnight. After reaction completion, the solvent was distilled under reduced pressure. The crude product was purified by column chromatography (*n*-hexane: ethyl acetate 1:1 *v:v*) to give 1.23 g of yellowish resin.

^{1}HNMR (400 MHz, $CDCl_3$): δ 9.23 (d, *J* = 8.5, 6.8, 1.4 Hz, 2H), 8.08 (dd, *J* = 8.1, 1.4 Hz, 2H), 7.96 (d, *J* =8.8 Hz, 2H), 7.71 (ddd, *J* =9.1, 7.8 Hz, 4H), 7.59 – 7.51 (m, 2H), 4.89 (ddd, *J* =9.7, 7.8, 5.5 Hz, 2H), 4.20 – 4.06 (m, 4H), 2.45 – 2.32 (m, 2H), 1.30 (t, *J* =7.1 Hz, 6H).

^{13}C NMR (101 MHz, $CDCl_3$): 136.5, 129.8, 129.2, 129.2, 126.9, 125.5, 125.1, 123.4, 117.6, 110.4, 62.0, 37.2, 27.1, 16.4.

Anal. calcd for $C_{26}H_{26}NO_3P$, %: C 72.38, H 6.07, N 3.25, found, %: C 72.21, H 6.29, N 3.24.

[2-(7H-dibenzo[c,g]carbazol-7-yl)ethyl]phosphonic acid (DBZ-2PACz, Figure S8C): Diethyl (2-(7*H*dibenzo[*c*,*g*]carbazol-7-yl)ethyl)phosphonate (2 mmol) was dissolved in anhydrous 1,4-dioxane (15 mL) under argon atmosphere and bromotrimethylsilane (23 mmol) was added dropwise. The reaction was stirred for 12 h at room temperature under an argon atmosphere. Afterwards, methanol was added and stirred for 3h. Finally, distilled water was added dropwise, until the solution became opaque, and stirred overnight. The product was filtered off, washed with water, dissolved in tetrahydrofuran and precipitated into *n*-hexane to give 0.67 g of white powder.

^{1}HNMR (400 MHz, $CDCl_3$): δ 9.06 (d, *J* = 8.5 Hz, 2H), 8.12 (d, *J* = 8.1 Hz, 2H), 8.07 – 7.93 (m, 4H), 7.71 (t, *J* = 7.6 Hz, 2H), 7.53 (t, *J* = 7.4 Hz, 2H), 4.93 – 4.81 (m, 2H), 3.89 (t, *J* = 7.3 Hz, 2H), 2.37 – 2.08 (m, 2H).

^{13}C NMR (101 MHz, $CDCl_3$): 136.29, 129.9, 129.7, 128.9, 127.1, 126.1, 124.6, 123.7, 116.7, 112.0, 60.64, 37.9.

Anal. calcd for $C_{22}H_{18}NO_3P$, %: C 70.40, H 4.83, N 3.735, found, %: C 70.59, H 4.58, N 3.74.

In the preparation of this manuscript, DBZ-2PACz was also synthesized by other groups [10]. Different from the reference using DBZ-2PACz for interfacial treatment at NiO_x-based tin-based perovskite, our study exploited the DBZ-2PACz directly in the lead-based PSCs due to its two advantages: reduced energy offset and strong interaction between 4-F-PEAFa and perovskite as well as the carrier transporter. These characteristics enable suppressed carrier transport losses at the perovskite adjacent interfaces.

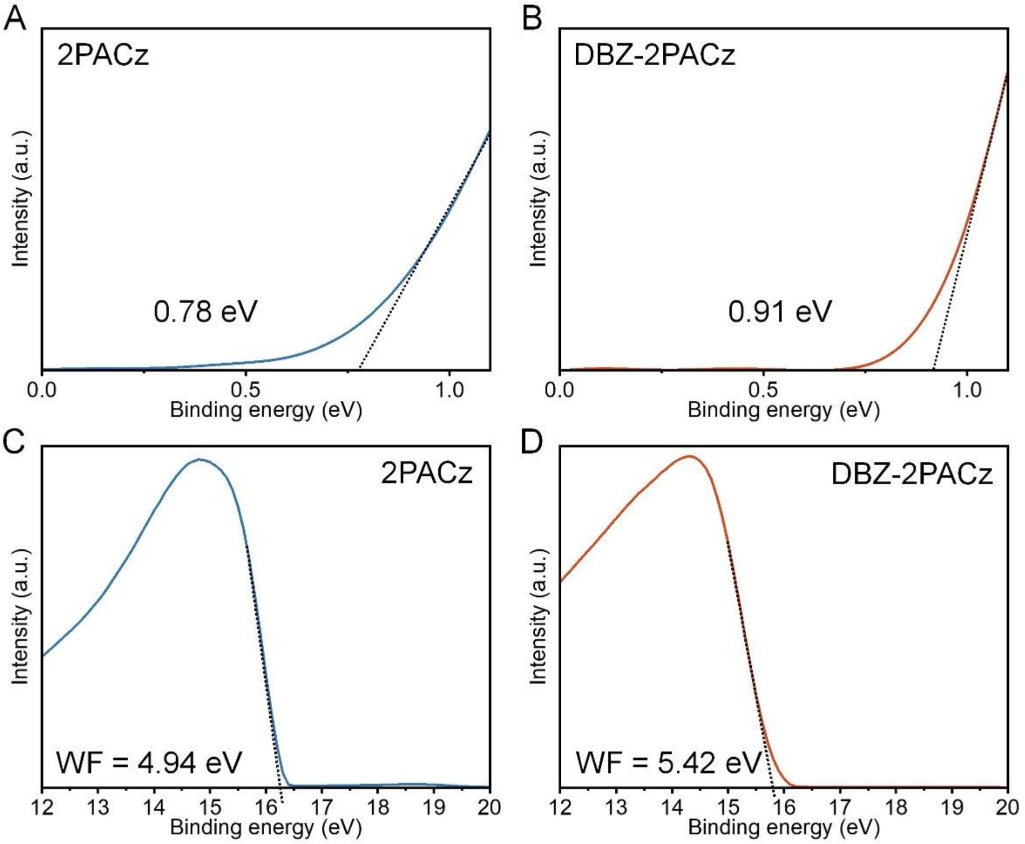

Figure S9. UPS spectra of 2PACz and DBZ-2PACz. (A,B) Valence band onset. The inset value denotes the energetic gap between the valence band maximum and Fermi level (set to 0 eV). (C,D) Secondary electron cutoff region. Both were fitted with a linear function and the intersection with the linear background was read as the work function and the valence band onset, respectively. The work function (WF) is determined as: $\mathrm{WF} = h\nu - E_0$, where $h\nu$ is the photon energy (21.22 eV) and E_0 is the intercept in the secondary electron cut-off.

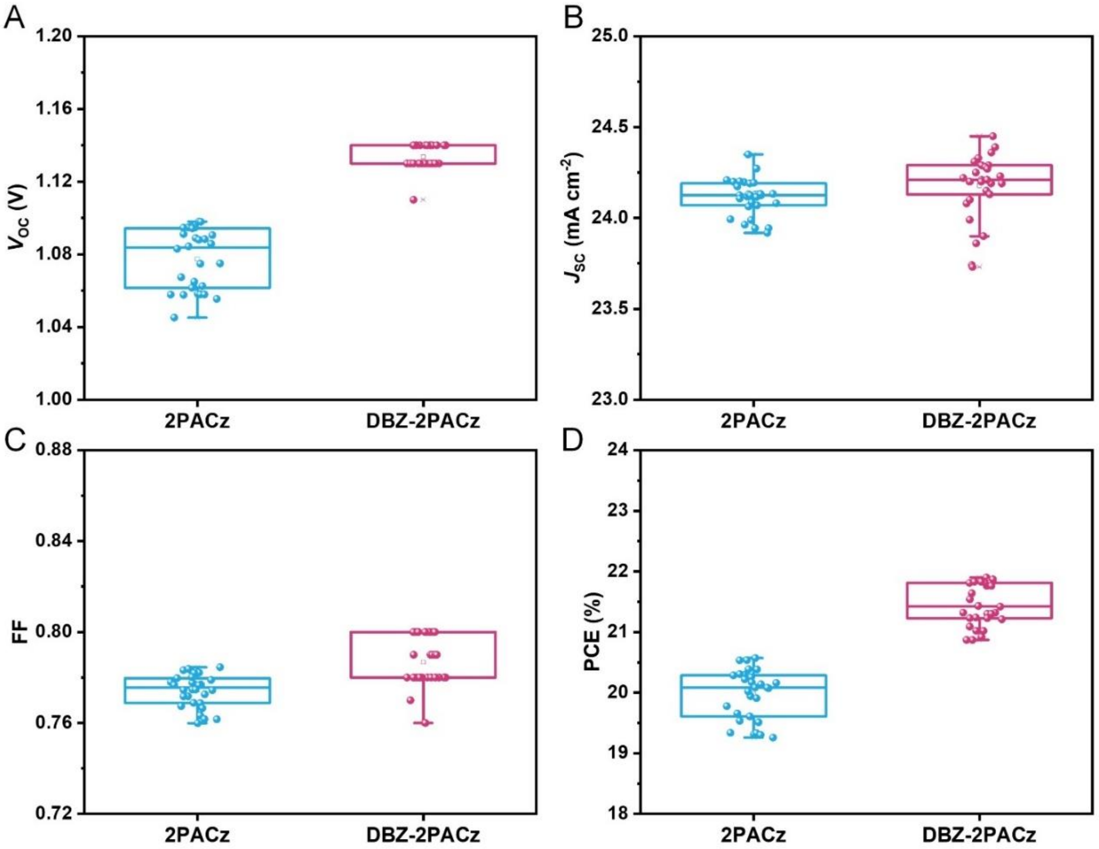

Figure S10. Statistical distributions of photovoltaic parameters (V_{OC}, J_{SC}, FF and PCE) based on the 2PACz and DBZ-2PACz. 30 devices were measured with a structure of glass/ITO/2PACz or DBZ-2PACz/perovskite/C_{60}/BCP/Cu, in which the perovskite is a control film (without additive).

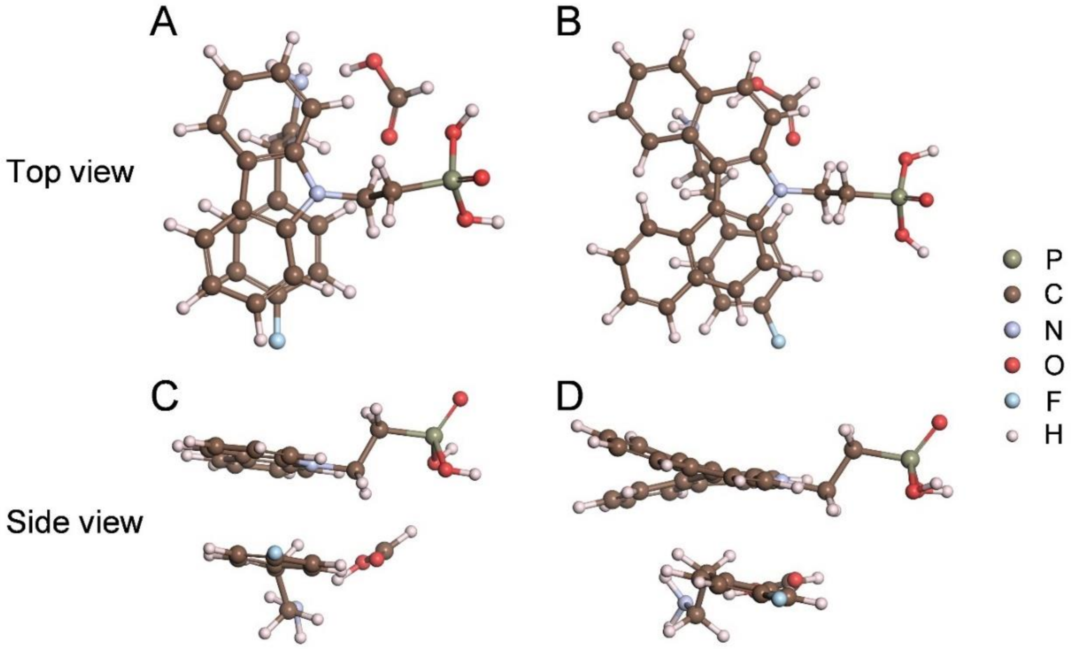

Figure S11. DFT-optimized structures of 4-F-PEAFa adsorbed with (A), (C) 2PACz and (B), (D) DBZ-2PACz.

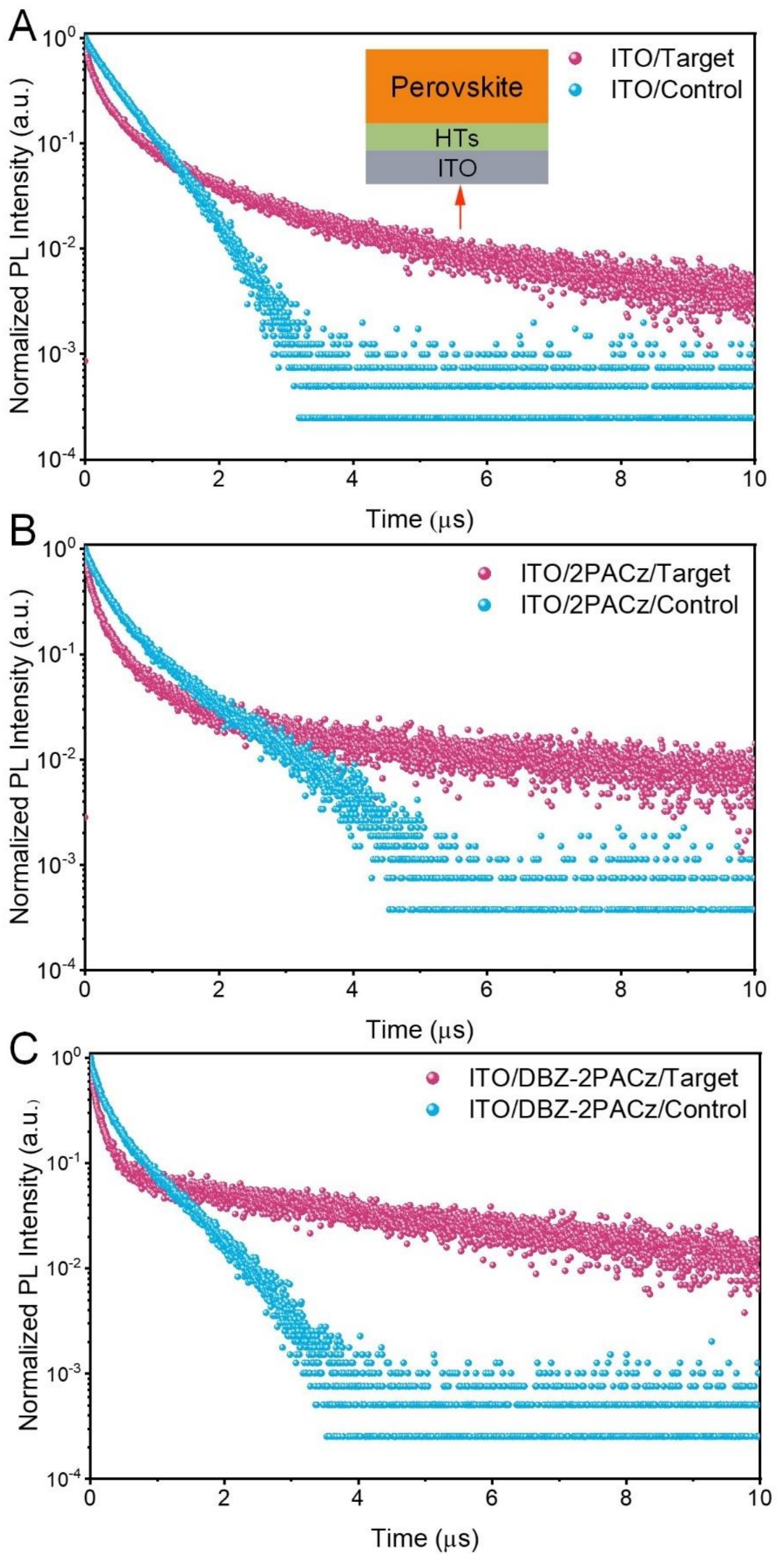

Figure S12. TRPL decay traces of perovskite film based on different hole transporters (HTs). The perovskite films were prepared with (target) or without (control) 4-F-PEAFa. All samples were measured with a 50 KHz repetition rate at a Fluence of ~24 nJ cm^{-2}. For the plots, the median number of counts before the laser absorption was subtracted from the data as background.

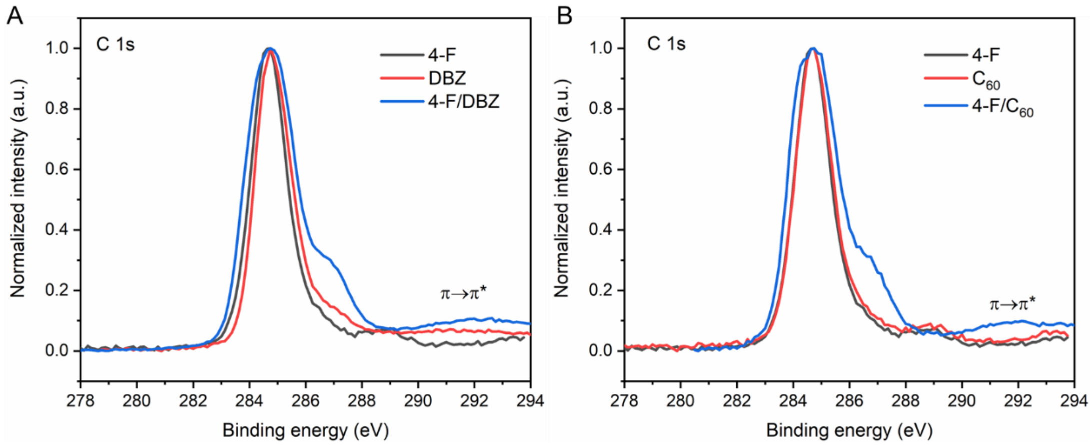

Figure S13. XPS spectra (C1s core-level energy) of the thin films, including (A) 4-F-PEAFa, DBZ-2PACz and their mixture. (B) 4-F-PEAFa, C_{60} and their mixture.

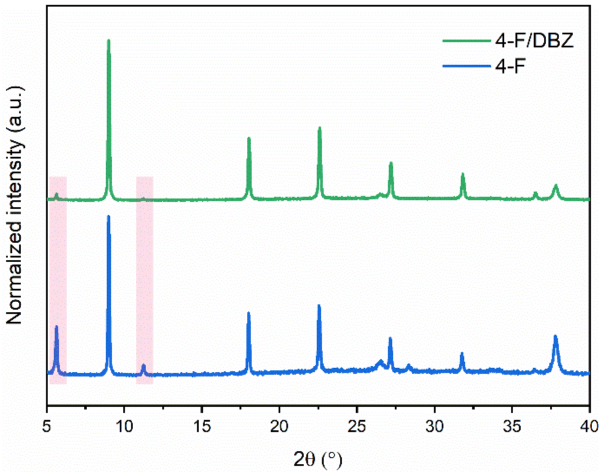

Figure S14. XRD patterns of thin-films made by DBZ-2PACz, 4-F-PEAFa and their mixture. All the films were spin-coated on FTO glass with the same program.

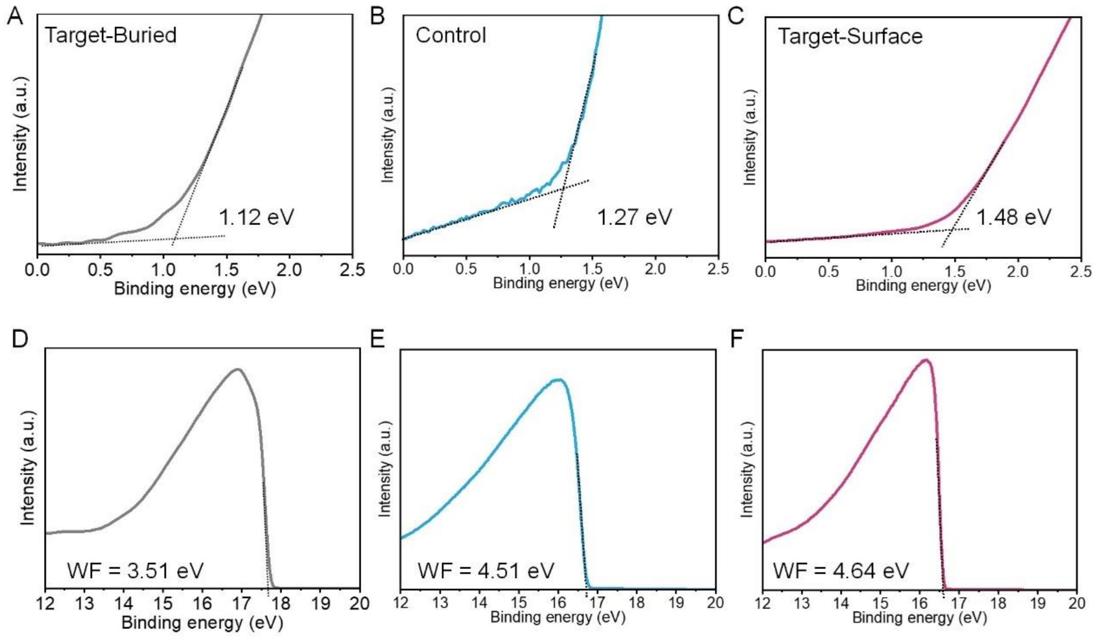

Figure S15. UPS spectra of perovskite film with (target) or without (control) 4-F-PEAFa. (A-C) Valence band onset. The inset value denotes the energetic gap between the valence band maximum and Fermi level (set to 0 eV). (D-F) Secondary electron cutoff region. Both were fitted with a linear function and the intersection with the linear background was read as the work function and the valence band onset, respectively. The work function (WF) is determined as: WF $= h\nu - E_0$, where $h\nu$ is the photon energy (21.22 eV) and E_0 is the intercept in the secondary electron cut-off. The detailed methodology for peeling the perovskite film for buried surface can be found in above film characterizations.

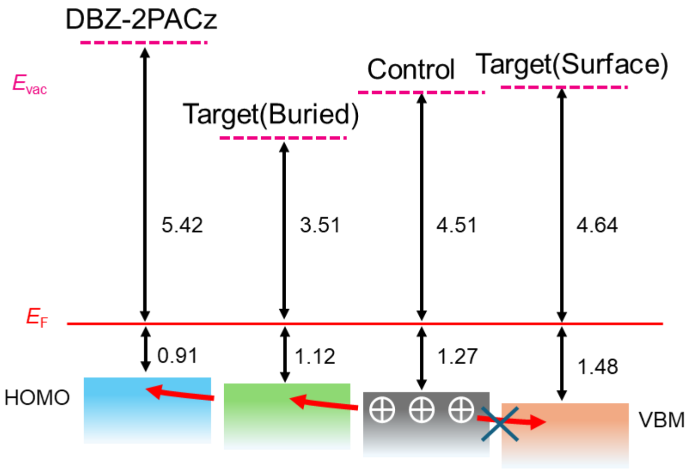

Figure S16. Band edge positions extracted from UPS measurements. E_{Vac} is vacuum level, E_F is Fermi level, HOMO is the highest occupied molecular orbital, and VBM is the valence band maximum.

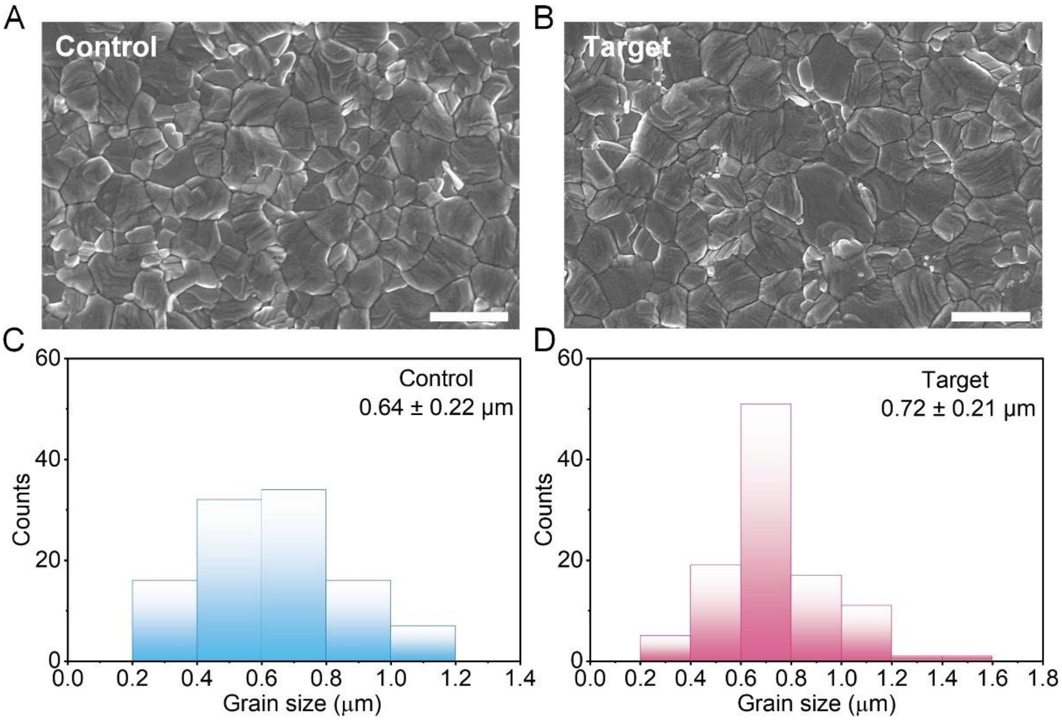

Figure S17. Top-view SEM images of perovskite films (A) without (control) or (B) with (target) 4-F-PEAFa. All films were deposited on the DBZ-2PACz-coated ITO glass. (C), (D) Corresponding distribution of grain sizes. Note that the scale bar in the SEM images is 1 μm and 105 grains of each condition were randomly selected for distribution analysis. The statistical analysis was performed on Jamovi software and the results of "*t*-test" (t (208) = 2.65, p = 0.009**) indicated a significant improvement in grain size based on the target.

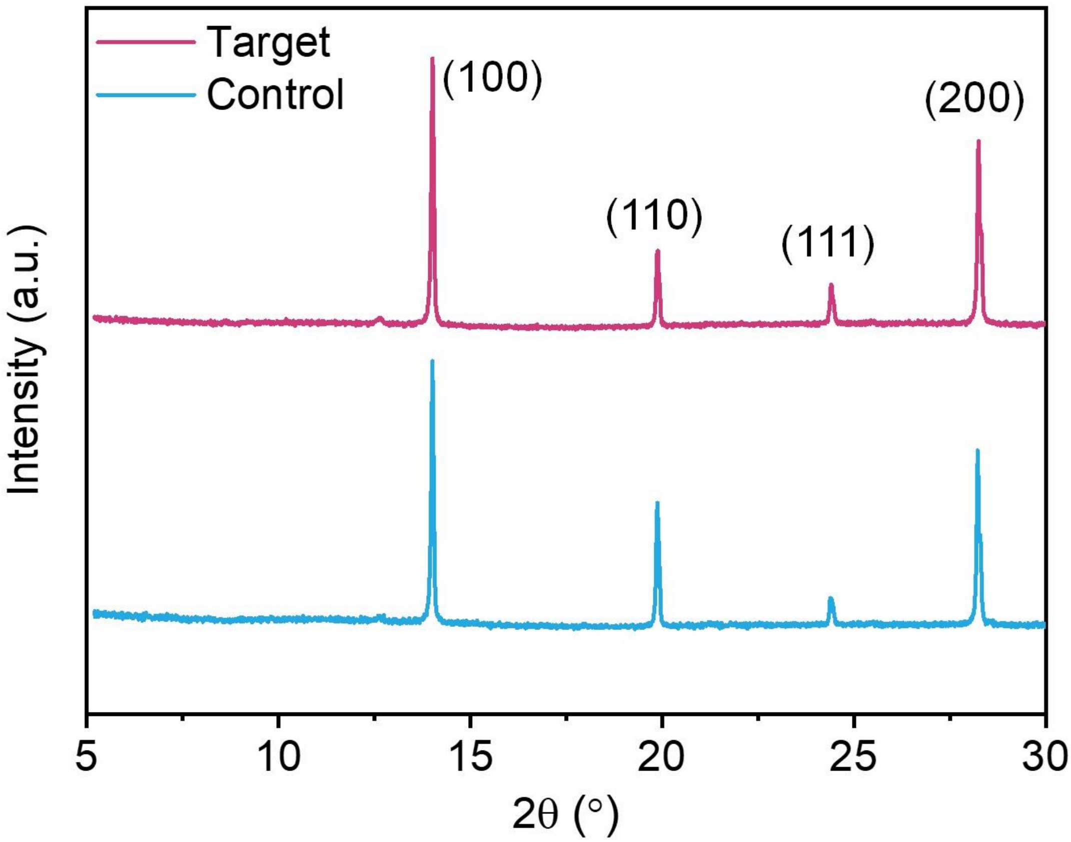

Figure S18. XRD pattern of perovskite films with (target) or without (control) 4-F-PEAFa. All films were deposited on the DBZ-2PACz-coated ITO glass.

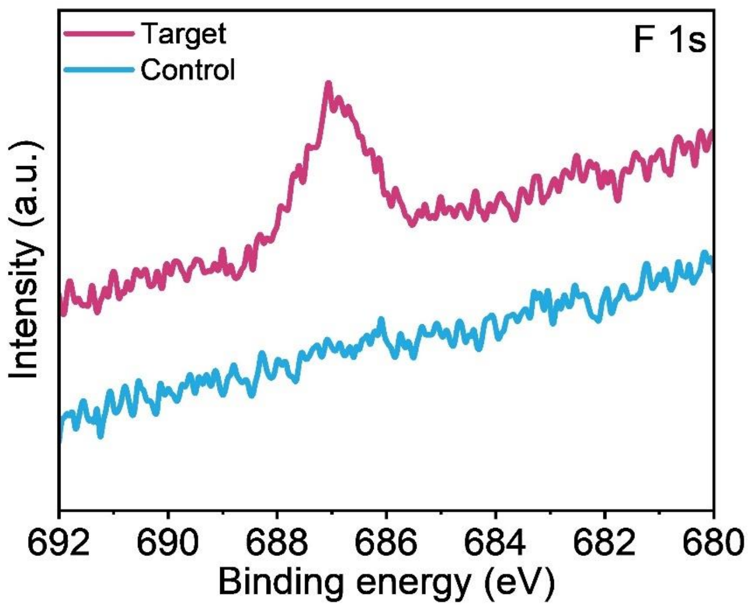

Figure S19. XPS spectra of F 1s based on the perovskite film with (target) or without (control) 4-F-PEAFa. The results confirmed the existence of 4-F-PEAFa on the perovskite surface.

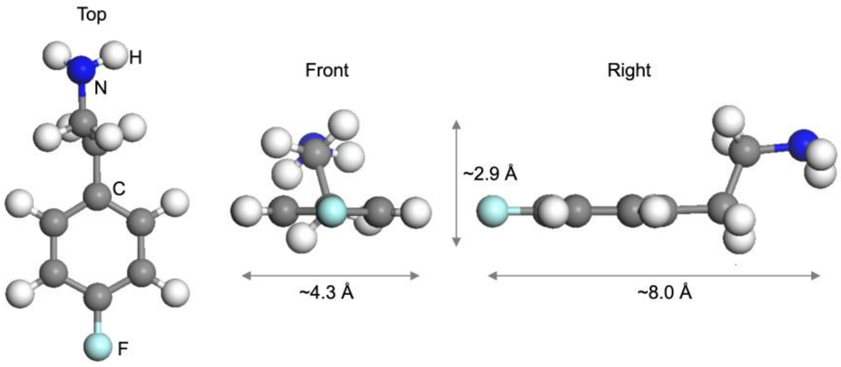

Figure S20. Optimized structure and dimensions of 4-F-PEA$^+$ from different view angels. Goldschmidt tolerance factor of perovskite (with a formula of ABX_3) can be calculated by using the following equation: $t = \frac{r_A + r_x}{\sqrt{2}(r_B + r_x)}$, where r_A, r_B, and r_X are the ionic radii for the ions in the A, B and X sites, respectively [11]. The ionic radii of FA^+, Pb^{2+} and I^- is 2.53 Å, 1.19 Å and 2.20 Å, respectively [12][13]. The exact ionic radius of 4-F-PEA$^+$ is hard to obtain due to the non-spherical geometry as shown in **Figure S19**, but we can use PEA^+ for comparison, which is reported as an oversized cation as the PEA has a conformal size of ~6 Å [14]. For FA-based perovskite ($FAPbI_3$):

$$t = \frac{r_A + r_x}{\sqrt{2}(r_B + r_x)} = \frac{2.53 + 2.20}{\sqrt{2}(1.19 + 2.20)} = 0.98$$

For 4-F-PEA-based perovskite (assuming 4-F-PEAPbI_3, using the minimum size of 2.9 Å for 4-F-PEA$^+$.):

$$t = \frac{r_A + r_x}{\sqrt{2}(r_B + r_x)} = \frac{2.9 + 2.20}{\sqrt{2}(1.19 + 2.20)} = 1.06$$

For most stable perovskites, *t* is ranging from 0.8 to 1 [15]. Hence, the calculated *t* for FA-based perovskite (0.98) falls within the stable perovskite range, confirming structural compatibility. Since 4-F-PEA$^+$ is obviously larger than FA^+, it likely exceeds the *t* range over 1. When $t > 1$, the structure becomes too distorted, and large A-site cations cannot fit the structural constraints of the lattice bulk [16], leading to surface accumulation. This supports the observation from ToF-SIMS that 4-F-PEA$^+$ accumulates near the surface rather than incorporating into the bulk perovskite.

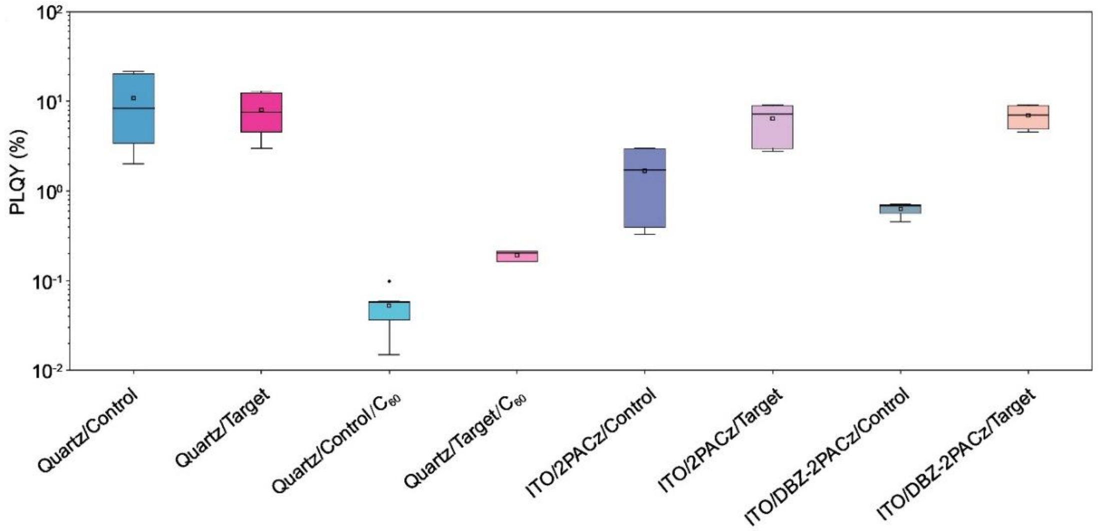

Figure S21. PLQY of the perovskite films when in contact with charge transport layers. Note that the perovskite is prepared with (target) or without (control) 4-F-PEAFa.

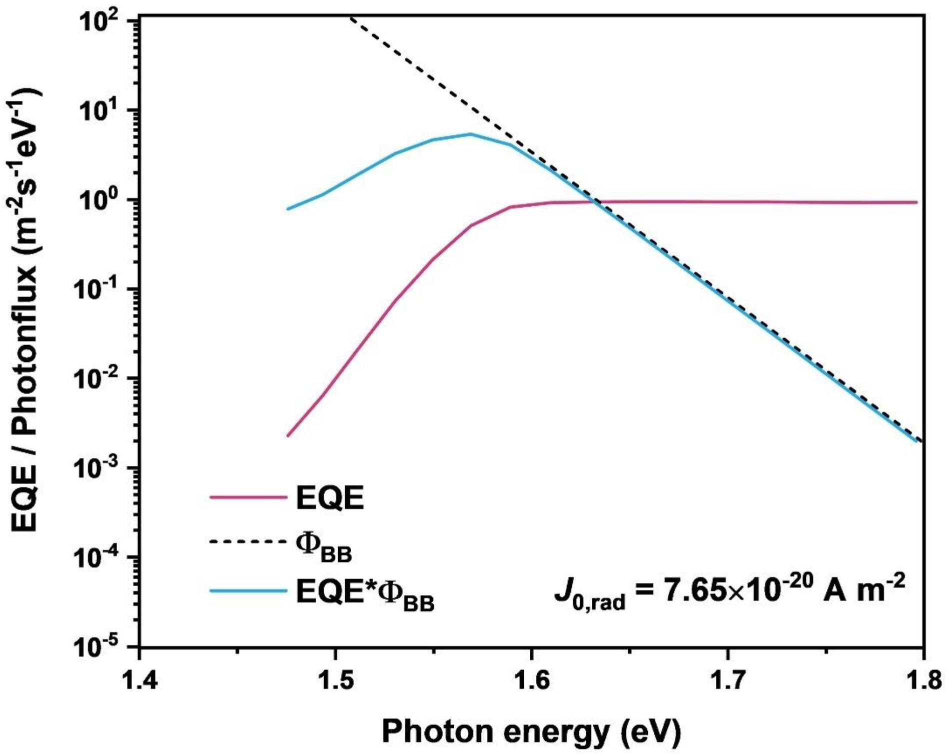

Figure S22. EQE_{PV} onset (red line) convoluted with the black-body (ϕ_{BB}) radiation of the surroundings at 300K (black dotted line).

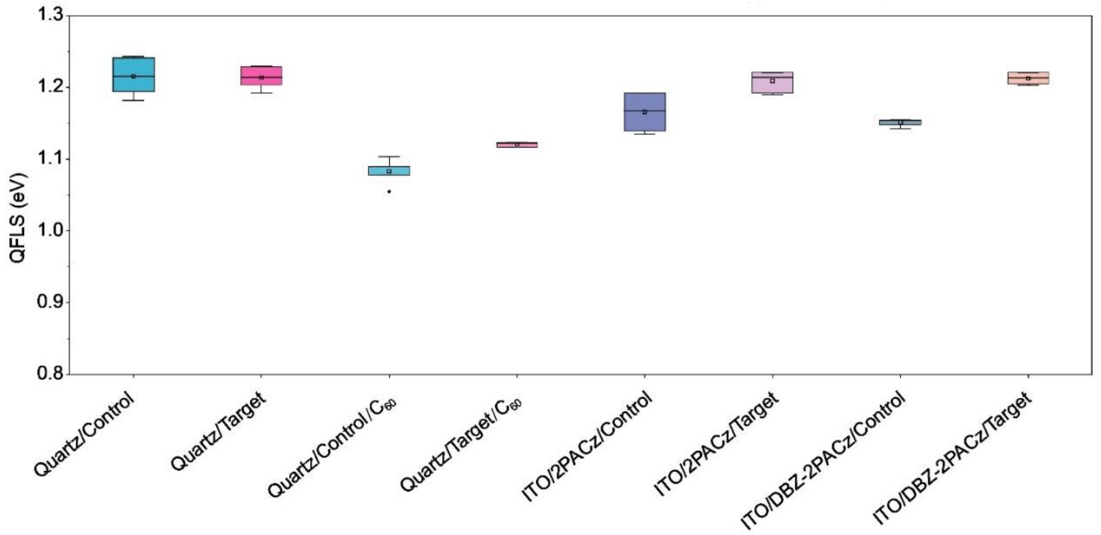

Figure S23. Calculated QFLS of the perovskite films when in contact with charge transporters. Note that the perovskite is prepared with (target) or without (control) 4-F-PEAFa.

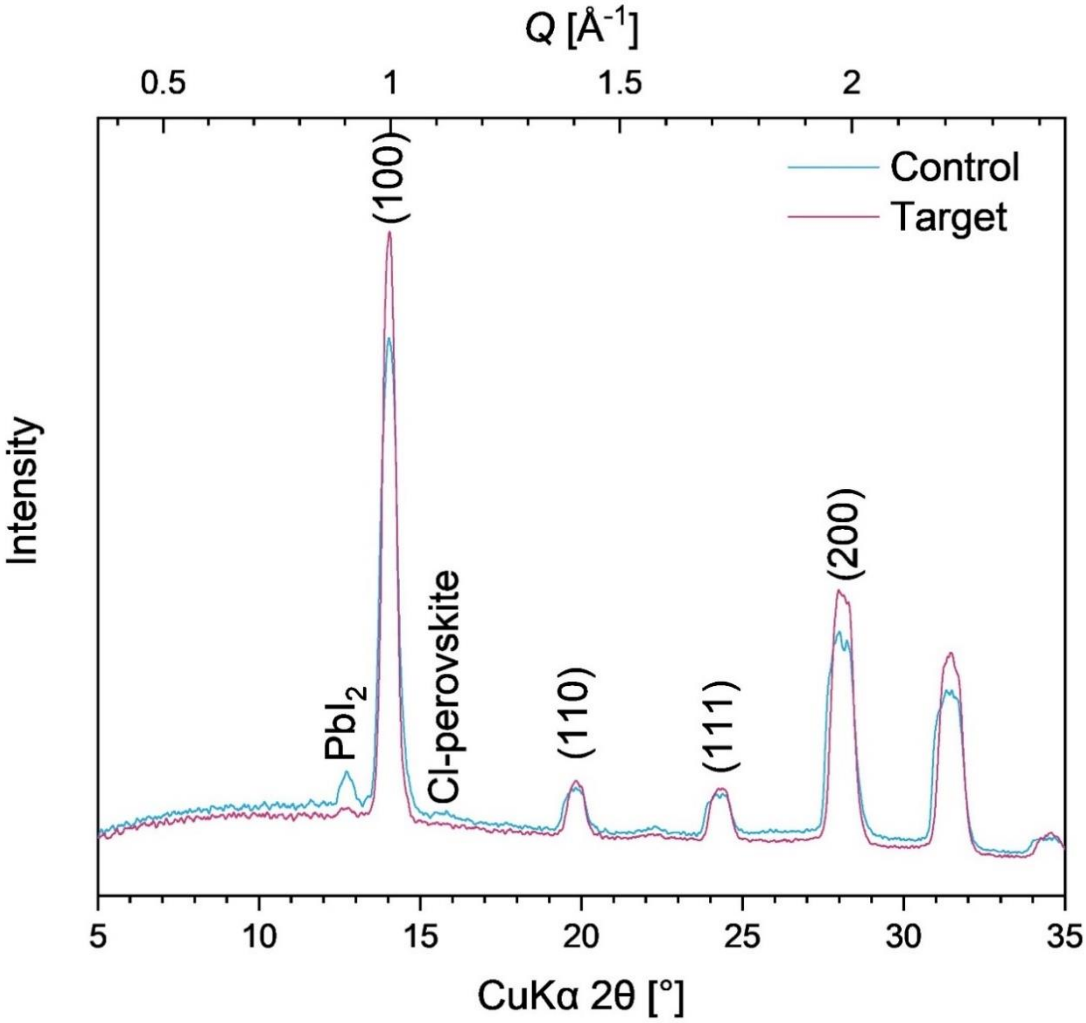

Figure S24. Azimuthally integrated 1D GIWAXS intensity from the 2D data in Figure 2C. Additional peaks correspond to PbI_2 and a trace amount of Cl-containing perovskite, both of which exhibit much lower intensity than the 4-F-PEAFa additive.

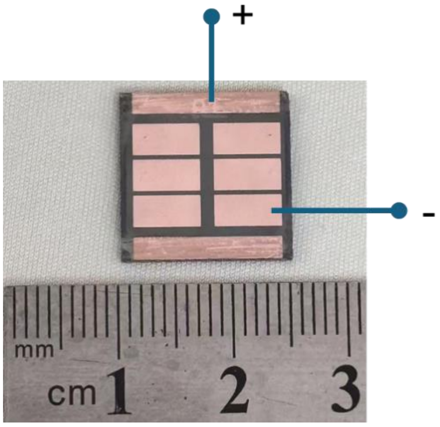

Figure S25. The photograph of the device with a structure of glass/ITO/DBZ-2PACz/perovskite/C_{60}/BCP/Cu.

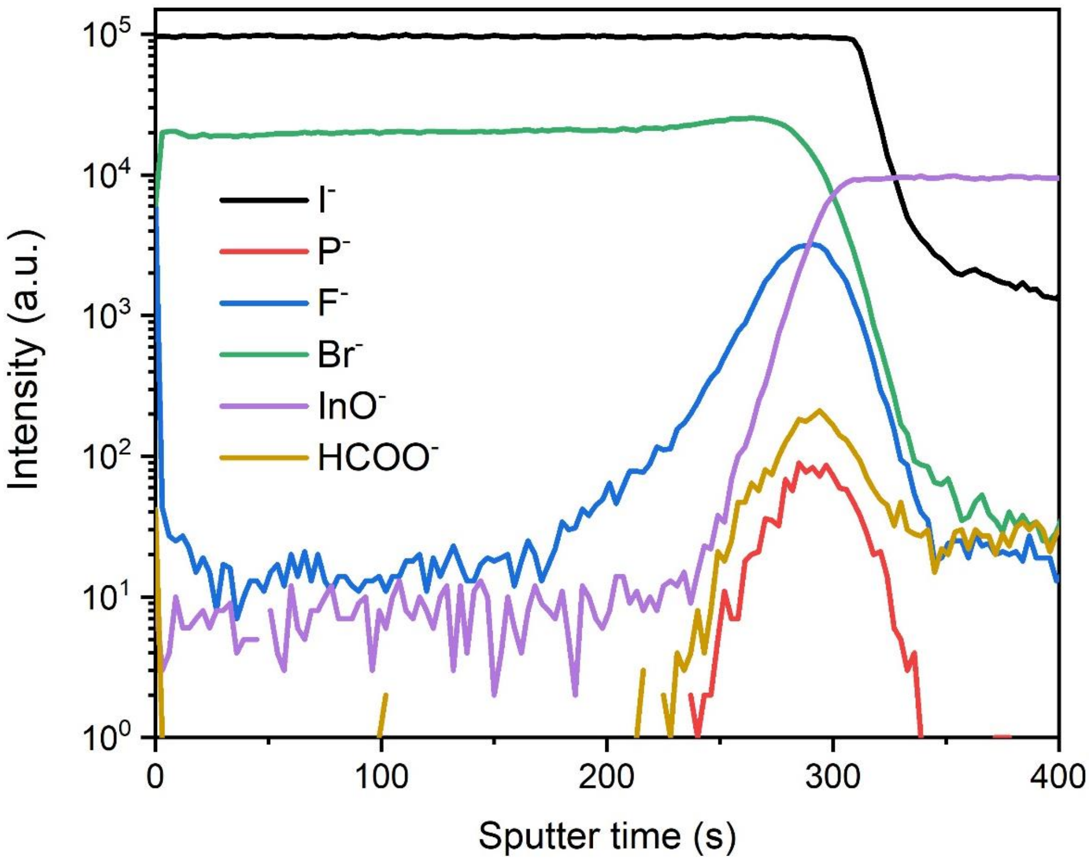

Figure S26. Depth profile of the negative ions from ToF-SIMS measurement. The 4-F-PEAFa-containing perovskite film is deposited on the glass/ITO/DBZ-2PACz same as the device fabrication. The P^- signal indicates the presence of DBZ-2PACz.[17]

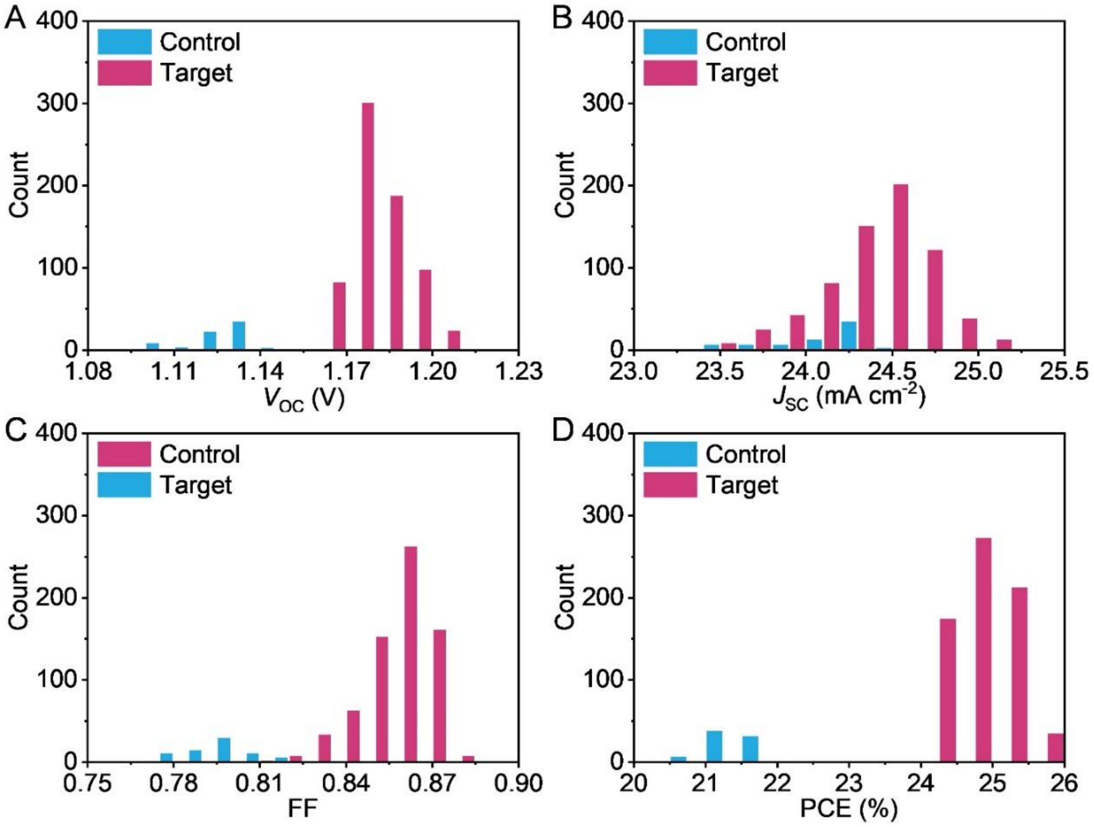

Figure S27. Histograms of photovoltaic parameters (V_{OC}, J_{SC}, FF and PCE) for control and target devices. 80 control devices and 700 target devices were measured, with a structure of glass/ITO/DBZ-2PACz/perovskite (with or without 4-F-PEAFa)/PDI/C_{60}/BCP/Cu. The devices were fabricated based on our optimized fabrication process and the extra anti-reflection layer (105 nm MgF_2) was evaporated on the glass side. Encouragingly, we noticed that some devices achieved the FF up to 0.88, which approaches the Shockley-Queisser (S-Q) limit of 0.903 at 1.57 eV bandgap [18].

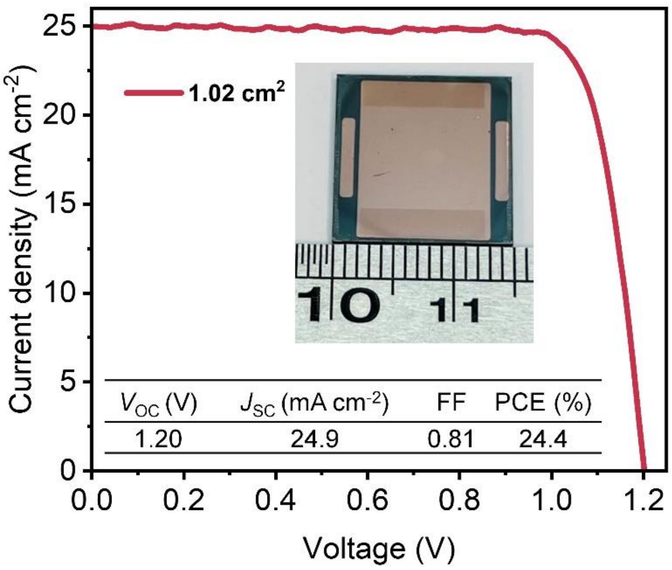

Figure S28. Champion performance of IPSCs based on an aperture area of 1.02 cm^2.

A

中国认可
国际互认
检测
TESTING
CNAS L8490

Test and Calibration Center of New Energy Device and Module,
Shanghai Institute of Microsystem and Information Technology,
Chinese Academy of Sciences (SIMIT)

Measurement Report

Report No. 23TR051801

**Client Name**	Chun Cheng's Group, Southern University of Science and Technology
**Client Address**	No. 1088, Xueyuan Avenue, Nanshan District, Shenzhen, China
**Sample**	Inverted Perovskite Solar Cell
**Manufacturer**	Chun Cheng's Group, Southern University of Science and Technology
**Measurement Date**	18th May, 2023

**Performed by:**	Qiang Shi *Qiang Shi*	**Date:** 18/05/2023
**Reviewed by:**	Wenjie Zhao *Wenjie Zhao*	**Date:** 18/05/2023
**Approved by:**	Zhengxin Liu *Zhengxin Liu*	**Date:** 22/05/2023

Address: No.235 Chengbei Road, Jiading, Shanghai **Post Code:**201800
E-mail: solarcell@mail.sim.ac.cn **Tel:** +86-021-69976921

The measurement report without signature and seal are not valid.
This report shall not be reproduced, except in full, without the approval of SIMIT.

B

Report No. 23TR051801

Sample Information

Sample Type	Inverted Perovskite Solar Cell
Serial No.	1-2#
Lab Internal No.	23051801-1#
Measurement Item	I-V characteristic
Measurement Environment	24.7±2.0°C,40.7±5.0%R.H

Measurement of I-V characteristic

**Reference cell**	PVM 1121
**Reference cell Type**	mono-Si, WPVS, calibrated by NREL (Certificate No. ISO 2075)
**Calibration Value/Date of Calibration for Reference cell**	144.53mA/ Feb. 2023
**Measurement Conditions**	Standard Test Condition (STC):<br>Spectral Distribution: AM1.5 according to IEC 60904-3 Ed.3,<br>Irradiance: 1000±50W/m^2, Temperature: 25±2°C
**Measurement Equipment/ Date of Calibration**	AAA Steady State Solar Simulator (YSS-T155-2M) / July.2022<br>IV test system (ADCMT 6246) / June. 2022<br>SR Measurement system (CEP-25ML-CAS) / April.2023<br>Measuring Microscope (MF-B2017C) / July.2022
**Measurement Method**	I-V Measurement:<br>Logarithmic sweep in both directions (Voc to Isc and Isc to Voc) during one flash based on IEC 60904-1:2020;<br>Spectral Mismatch factor was calculated according to IEC 60904-7 and I-V correction according to IEC 60891;
**Measurement Uncertainty**	Area: 1.0%(k=2); Isc: 1.8%(k=2); Voc: 1.0%(k=2); Pmax: 2.3%(k=2); Eff: 2.5%(k=2)

C

Report No. 23TR051801

====Measurement Results ====

	Forward Scan (Isc to Voc)	Reverse Scan (Voc to Isc)
Area	8.49 mm^2	
Isc	2.130 mA	2.130 mA
Voc	1.172 V	1.172 V
Pmax	2.135 mW	2.170 mW
Ipm	2.039 mA	2.047 mA
Vpm	1.047 V	1.060 V
FF	85.53 %	86.93 %
Eff	25.15 %	25.56 %

- Spectral Mismatch Factor: SMM=0.9921.
- Designated illumination area defined by a thin metal mask was measured by the measuring microscope.
- Test results listed in this measurement report refer exclusively to the mentioned measured sample.
- The results apply only at the time of the test, and do not imply future performance.

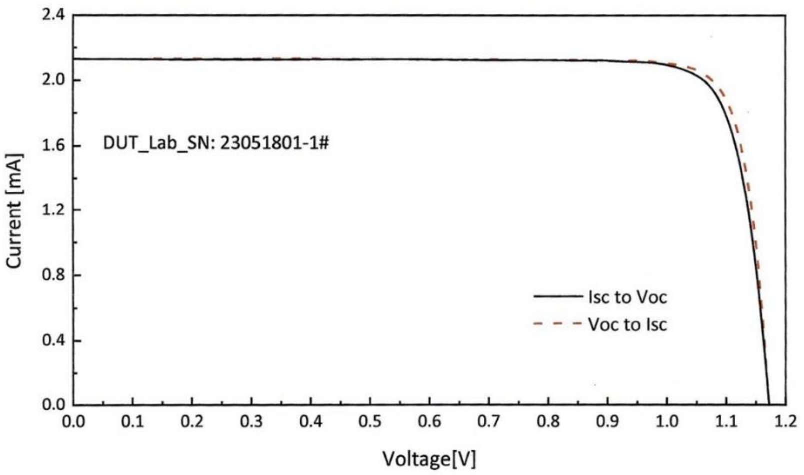

Fig.1 I-V curves of the measured sample

-------End of Report-------

D

中国认可
国际互认
检测
TESTING
CNAS L8490

Test and Calibration Center of New Energy Device and Module,
Shanghai Institute of Microsystem and Information Technology,
Chinese Academy of Sciences (SIMIT)

Measurement Report

Report No. 23TR051803

**Client Name**	Chun Cheng's Group, Southern University of Science and Technology
**Client Address**	No. 1088, Xueyuan Avenue, Nanshan District, Shenzhen, China
**Sample**	Inverted Perovskite Solar Cell
**Manufacturer**	Chun Cheng's Group, Southern University of Science and Technology
**Measurement Date**	18th May, 2023

**Performed by:**	Qiang Shi Qiang Shi	**Date:** 18/05/2023
**Reviewed by:**	Wenjie Zhao Wenjie Zhao	**Date:** 18/05/2023
**Approved by:**	Zhengxin Liu Zhengxin Liu	**Date:** 22/05/2023

Address: No.235 Chengbei Road, Jiading, Shanghai **Post Code:**201800
E-mail: solarcell@mail.sim.ac.cn **Tel:** +86-021-69976921

The measurement report without signature and seal are not valid.
This report shall not be reproduced, except in full, without the approval of SIMIT.

E

Sample Information

Sample Type	Inverted Perovskite Solar Cell
Serial No.	23-1#
Lab Internal No.	23051801-3#
Measurement Item	I-V characteristic
Measurement Environment	24.7±2.0°C,40.7±5.0%R.H

Measurement of I-V characteristic

**Reference cell**	PVM 1121
**Reference cell Type**	mono-Si, WPVS, calibrated by NREL (Certificate No. ISO 2075)
**Calibration Value/Date of Calibration for Reference cell**	144.53mA/ Feb. 2023
**Measurement Conditions**	Standard Test Condition (STC):<br>Spectral Distribution: AM1.5 according to IEC 60904-3 Ed.3,<br>Irradiance: 1000±50W/m^2, Temperature: 25±2°C
**Measurement Equipment/ Date of Calibration**	AAA Steady State Solar Simulator (YSS-T155-2M) / July.2022<br>IV test system (ADCMT 6246) / June. 2022<br>SR Measurement system (CEP-25ML-CAS) / April.2023<br>Measuring Microscope (MF-B2017C) / July.2022
**Measurement Method**	I-V Measurement:<br>Logarithmic sweep in both directions (Voc to Isc and Isc to Voc) during one flash based on IEC 60904-1:2020;<br>Spectral Mismatch factor was calculated according to IEC 60904-7 and I-V correction according to IEC 60891;
**Measurement Uncertainty**	Area: 1.0%(k=2); Isc: 1.8%(k=2); Voc: 1.0%(k=2); Pmax: 2.3%(k=2); Eff: 2.5%(k=2)

F

Report No. 23TR051803

====**Measurement Results** ====

	Forward Scan (Isc to Voc)	Reverse Scan (Voc to Isc)
Area	8.49 mm^2	
Isc	2.115 mA	2.117 mA
Voc	1.174 V	1.174 V
Pmax	2.122 mW	2.173 mW
Ipm	2.029 mA	2.042 mA
Vpm	1.046 V	1.064 V
FF	85.51 %	87.46 %
Eff	25.00 %	25.59 %

- Spectral Mismatch Factor: SMM=0.9921.
- Designated illumination area defined by a thin metal mask was measured by the measuring microscope.
- Test results listed in this measurement report refer exclusively to the mentioned measured sample.
- The results apply only at the time of the test, and do not imply future performance.

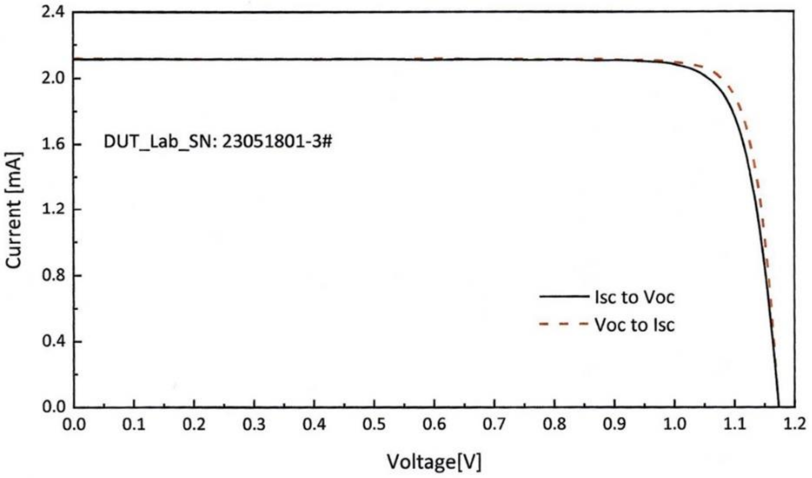

Fig.1 I-V curves of the measured sample

-------**End of Report**-------

Figure S29. Independent efficiency certifications of device 1 (A, B, C) and device 4 (D, E, F). Note that more certified data are summarized in Table S4.

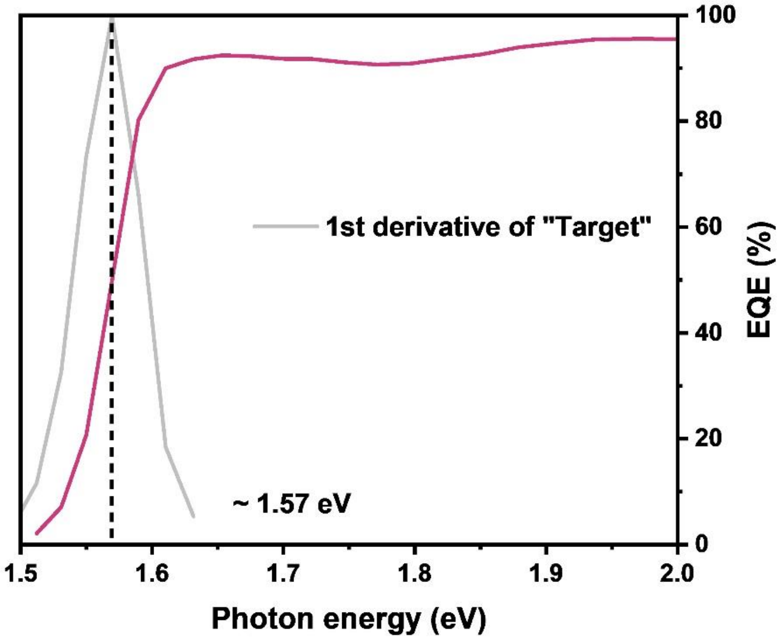

Figure S30. Derivative of EQE spectrum to identify the bandgap (E_g =1.57 eV) of the perovskite.

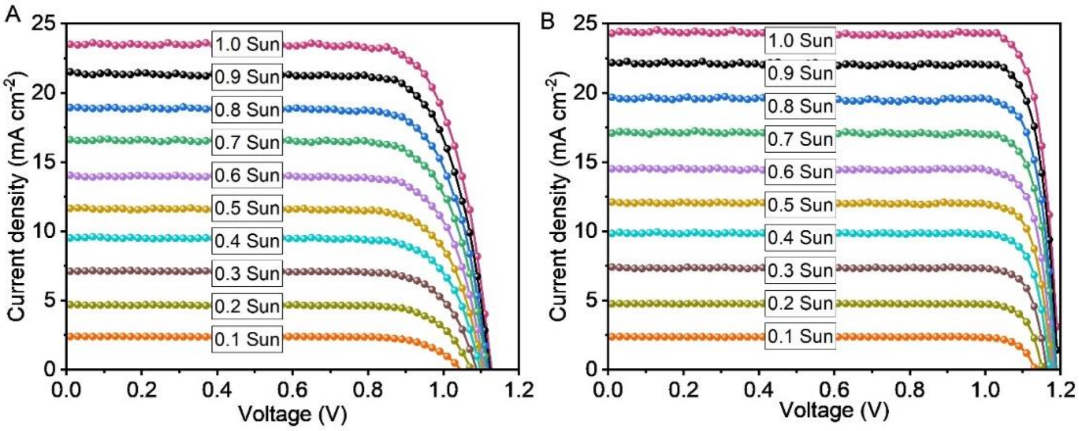

Figure S31. *J-V* curves measured under light intensities ranging from 0.1 to 1 Sun**.** (A) Control and (B) Target devices, with a structure of glass/ITO/DBZ-2PACz/perovskite (with or without 4-F-PEAFa)/PDI/C_{60}/BCP/Cu. The control and target devices are randomly selected, with initial V_{OC} and FF of 1.13 V, 0.77 and 1.20 V, 0.87, which were used in the Note S7.

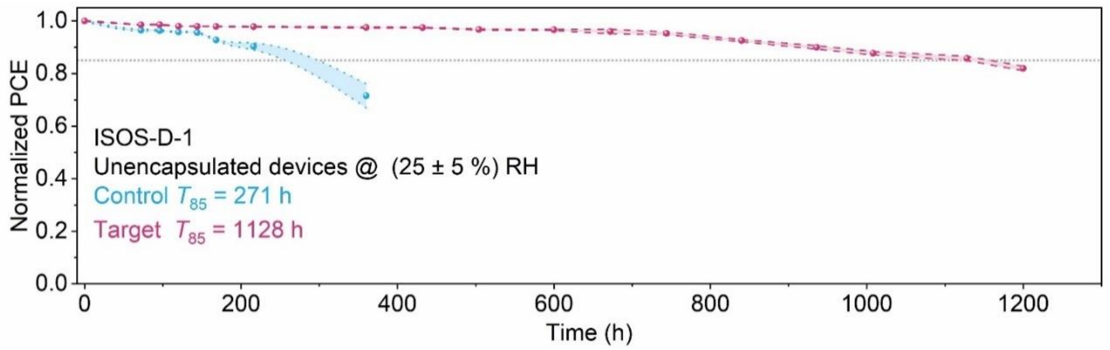

Figure S32. Shelf-life stability in air (equivalent to ISOS-D-1). 5 devices were measured for each condition with the estimated T_{85} (lifetime at 85% of the initial PCEs).

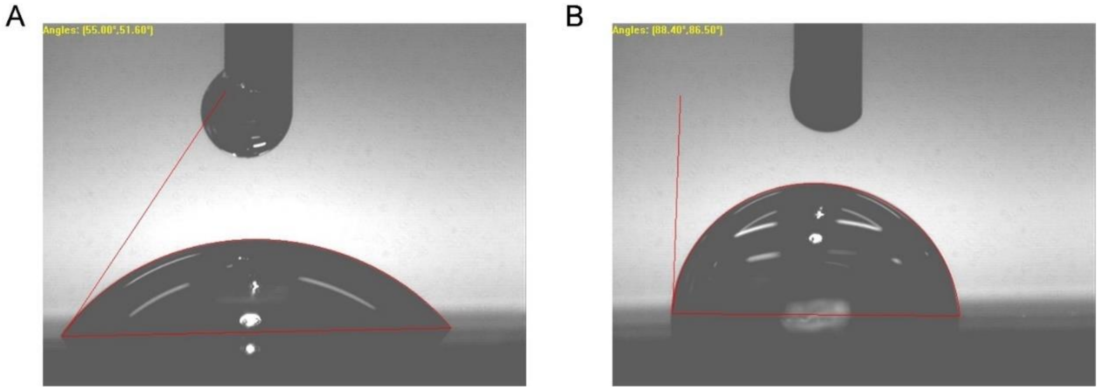

Figure S33. Contact angle of (A) control and (B) target perovskite film. A larger contact angle was shown on the target film (88.4°) than the control film (55°), indicating the enhanced hydrophobicity of the perovskite film.

Table S1. Summary of high-performance IPSCs (exceeding 24% PCEs, certified), consisting of the photovoltaic parameters, ideality factor (n_{id}), *f* (FF relative to its S-Q limit) and *m* (V_{OC} × FF relative to its S-Q limit). Note that the PVK denotes perovskite. AA denotes aperture area. The asterisk indicates certified values. The S-Q limit of each photovoltaic parameter is obtained from a cell illuminated by the AM 1.5G spectral irradiance (ASTM G173-03) at 25 °C [18]. The certified device in this work yielded the highest FF and *f* amongst all IPSCs.

Device structure	E_g (eV)	V_{OC} (V)	J_{SC} (mA cm^{-2})	FF	PCE (%)	AA (cm^2)	*f*	*m*	n_{id}	Ref.
ITO/PTAA/PVK/C_{60}/BCP/Cu	1.55	1.18*	25.6*	0.81*	24.3*	0.0812*	0.90	0.84	/	[19]
ITO/MeO-2PACz/PVK/PCBM/BCP/Cu	1.55	1.17*	24.8*	0.83*	24.2*	0.0958*	0.92	0.85	/	[20]
ITO/MeO-2PACz/PVK/C_{60}/BCP/Cu	1.53	1.16*	26.1*	0.84*	25.4*	0.0583*	0.93	0.87	/	[21]
ITO/Me-4PACz/PVK/C_{60}/BCP/Ag	1.55	1.20*	25.0*	0.83*	24.9*	0.0583*	0.92	0.87	/	[22]
ITO/DC-PA/PVK/C_{60}/BCP/Cu	1.55	1.19*	24.5*	0.84*	24.5*	0.0400*	0.93	0.88	/	[23]
ITO/PVK/PCBM/BCP/Ag	1.56	1.19*	25.7*	0.83*	25.4*	0.0802*	0.92	0.86	/	[24]
ITO/2PACZ:MPA/PVK/PCBM/BCP/Ag	1.54	1.15*	25.5*	0.85*	24.8*	0.0494*	0.94	0.87	/	[25]
FTO/NiO_x/Me-4PACz/PVK/C_{60}/BCP/Ag	1.50	1.17*	25.7*	0.83*	25.1*	0.04943*	0.92	0.89	/	[26]
FTO/NiO_x/Me-4PACz/PVK/PCBM/SnO_2/Cu	1.52	1.18*	26.2*	0.81*	25.2*	0.07356*	0.90	0.86	1.22	[27]
ITO/NiO_x/MeO-4PADBC/PVK/C_{60}/BCP/Ag	1.53	1.19*	25.7*	0.84*	25.6*	0.0414*	0.93	0.89	1.55	[28]
ITO/PTAA/PVK/C_{60}/BCP/Ag	1.52	1.15*	26.5*	0.85*	25.8*	0.0749*	0.94	0.88	/	[29]
FTO/Me-4PACz/PVK/C_{60}/BCP/Cu	1.55	1.18*	25.4*	0.86*	25.9*	0.075*	0.95	0.89	/	[30]
ITO/ATO_x/Me-4PACz/PVK/C_{60}/BCP/Cu	1.55	1.17*	25.7*	0.84*	25.1*	0.05012*	0.93	0.86	/	[31]
FTO/MeO-2PACz/PVK/PCBM/YbO_x/Cu	1.54	1.16*	25.8*	0.84*	25.1*	0.07221*	0.93	0.86	/	[32]
ITO/2PACz/PVK/C_{60}/BCP/Ag	1.54	1.17*	25.0*	0.86*	25.0*	0.0626*	0.93	0.88	/	[33]
ITO/2PACz/PVK/C_{60}/BCP/Cu	1.56	1.17*	25.3*	0.85*	25.3*	0.0680*	0.93	0.87	/	[34]
FTO/Me-4PACz/PVK/C_{60}/SnO_2/Ag	1.53	1.17*	26.1*	0.85*	26.15*	0.04929*	0.94	0.89	/	[35]
ITO/NiO_x/ Me-4PACz/PVK/PCBM/BCP/Ag	1.53	1.20*	26.5*	0.84*	26.5*	0.057*	0.97	0.90	/	[36]
FTO/MeO-2PACz/PVK/PCBM/BCP/Ag	1.54	1.17*	26.3*	0.85*	25.9*	0.07221*	0.93	0.87	/	[37]
ITO/Py3/PVK/LiF/C_{60}/PVK/Ag	1.53	1.19*	25.7*	0.84*	25.7*	0.048*	0.95	0.89	/	[38]
ITO/A1090B769/ PVK/PCBM/BCP/Ag	1.55	1.20*	26.1*	0.82*	25.9*	0.0778*	0.95	0.87	/	[39]
ITO/2PACz/TATPA/ PVK/C_{60}/BCP/Ag	1.55	1.19*	26.3*	0.84*	26.1*	0.101*	0.94	0.87	/	[40]
ITO/PTAA/MoS_2/ PVK/MoS_2/C_{60}/BCP/Ag	1.52	1.20*	25.9*	0.84*	26.2*	0.07393*	0.95	0.89	/	[41]
**ITO/DBZ-2PACz/PVK/C_{60}/BCP/Cu**	**1.57**	**1.17***	**25.1***	**0.87***	**25.6***	**0.0849***	**0.96**	**0.88**	**1.06**	**This work**

Table S2. DFT-predicted electrostatic potential energy (φ) mapped on charge density isosurface of *n*-F-PEA, dipole moment (μ) of *n*-F-PEA, dimerization energy (E_{dimer}) of *n*-F-PEA, and V_{I} formation energy (E_f) in the *n*-F-PEAI-terminated surface.

Species	φ (eV)	μ (Debye)	E_{dimer} (eV)	E_f (eV)
2-F-PEA	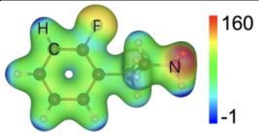	7.28	-0.15	2.06
3-F-PEA	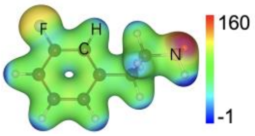	8.26	-0.17	2.15
4-F-PEA	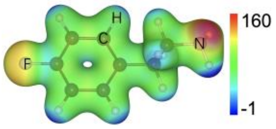	9.00	-0.19	2.35

Table S3. Dissociation energy of molecules in the N, N-dimethyl-formamide solution predicted by DFT calculations. The stability order is 4-F-PEAI > FAI > FACOOH; $Pb(HCOO)_2$ > PbI_2.

Dissociation reaction	ΔE (eV)
FACOOH → FA^+ + $HCOO^-$	0.54
FAI → FA^+ + I^-	0.63
4-F-PEAI → 4-F-PEA^+ + I^-	0.68
4-F-PEACOOH → 4-F-PEA^+ + $HCOO^-$	0.81
PbI_2 → Pb^{2+} + $2I^-$	4.22
$Pb(HCOO)_2$ → Pb^{2+} + $2HCOO^-$	4.44

Table S4. Certified data of 5 devices, including the forward scan (FS) and reverse scan (RS). The certification reports of device 1 and device 4 are displayed in Figure S29. Note that encapsulation processing was not utilized for any of the devices during the certification process. The average values denote arithmetic mean ± standard deviation.

Device	Scan	V_{OC} (V)	J_{SC} (mA cm^{-2})	FF	PCE (%)
1	FS	1.17	25.1	0.855	25.2
	RS	1.17	25.1	0.869	25.6
2	FS	1.17	24.8	0.857	24.8
	RS	1.17	24.8	0.873	25.3
3	FS	1.17	24.6	0.855	24.6
	RS	1.17	24.6	0.870	25.0
4	FS	1.17	24.9	0.855	25.0
	RS	1.17	24.9	0.875	25.6
5	FS	1.17	24.6	0.859	24.8
	RS	1.17	24.6	0.877	25.4
**Average**		**1.17**	**24.8 ± 0.2**	**0.866 ± 0.01**	**25.1 ± 0.35**

Note S1. Computational details.

Density functional theory (DFT) calculations were performed using the Vienna *Ab-initio* Simulation Package.[42] The generalized gradient approximation in the Perdew-Burke-Ernzerhof form was adopted as the exchange-correlation functional.[43] The van der Waals interactions were included using the DFT-D3 dispersion correction method.[44] A plane-wave energy cutoff of 505 eV was set together with the following projector-augmented wave pseudopotentials:[45] C_GW ($2s^2 2p^2$) for carbon, N_GW ($2s^2 2p^3$) for nitrogen, O_GW ($2s^2 2p^4$) for oxygen, F_PBE ($2s^2 2p^5$) for fluorine, H_GW ($1s^1$) for hydrogen, Br_PBE ($4s^2 4p^5$) for bromine, I_GW ($5s^2 5p^5$) for iodine, Pb_PBE ($6s^2 6p^2$) for lead, and Cs_GW ($4d^2 5p^6 6s^1$) for cesium. A Monkhorst-Pack mesh with a spacing of ~2π/30 $Å^{-1}$ was adopted for the Brillouin zone sampling. The energy and force convergence tolerance were 10^{-5} eV and 0.01 eV $Å^{-1}$, respectively. The defect formation energy and binding energy calculations were based on a slab supercell of $Cs_4FA_{68}MA_8Pb_{80}I_{232}Br_8$ (~26.04 × 25.82 × 29.46 $Å^3$) with a vacuum layer of ~16 Å in the out-of-plane direction. The dimerization energy of *n*-F-PEA is defined as follows:

$$E_{dimer} = E(dimer) - 2E(monomer) \quad \text{(S1)}$$

where *E*(*dimer*) and *E*(*monomer*) are the total energies of the *n*-F-PEA dimer and monomer, respectively. The formation energy E_f of iodine vacancy V_I in the FAI- or *n*-F-PEAI-terminated surface was calculated as follows:

$$E_f(V_I) = E_s(V_I) + \mu_I - E_s(perfect) \quad \text{(S2)}$$

where $E_s(V_I)$ and $E_s(perfect)$ are the total energies of a surface with and without V_I, respectively; μ_I is the total energy of iodine bulk per atom. The binding energies between surface defects and various anions were calculated using the following equation:

$$E_b = E_s(anion) - E_s(defect) - E_{anion} \quad \text{(S3)}$$

where $E_s(defect)$ is the total energy of a surface structure with defect V_I, Pb_I, or FA_i; $E_s(anion)$ is the total energy of the surface with defect passivated by anion I^-, Br^-, Cl^-, or $HCOO^-$; E_{anion} is the total energy of anion.

The dissociation energy of molecules and the binding energy between 4-F-PEAFa and DBZ-2PACz, 2PACz, or C_{60} were calculated using the Gaussian 16 package with the B3LYP functional and the DFT-D3 correction.[46] The basis sets were 6-311++G** for C, H, N, and O and def2-SVP for Pb and I.

Note S2. The grain-size differences between the control and the target perovskite film.

We conducted the significance test to examine the grain-size differences between the control and the target. We first investigated the data normality.

	**Skewness**	**SE$_{\text{skewness}}$**	**Z$_{\text{skewness}}$**	**Kurtosis**	**SE$_{\text{kurtosis}}$**	**Z$_{\text{kurtosis}}$**
**Target**	0.590	0.241	2.45	1.45	0.467	3.10
**Control**	0.334	0.241	1.38	–0.459	0.467	–0.983

The significance of skewness or kurtosis can be determined by the Z-scores, defined by $Z_{\text{skewness}} = \frac{\text{skewness}}{\text{SE}_{\text{skewness}}}$ or $Z_{\text{kurtosis}} = \frac{\text{kurtosis}}{\text{SE}_{\text{kurtosis}}}$, wherein $\text{SE}_{\text{skewness}}$ and $\text{SE}_{\text{kurtosis}}$ denote the standard error of skewness and standard error of kurtosis, respectively. For medium-sized samples (N > 100), the Z-scores below – 3.29 or above 3.29 indicate significant skewness or kurtosis. Here, the grain sizes of both groups are non-significant. Also, the assumption about the homogeneity of variances is met ($p = 0.248$). Therefore, the "*t*-test" is used to examine the data, in which *t* is the "*t*-test" score, *df* denotes the degrees of freedom, *p* is the probability under the null hypothesis and *d* represents the effect size.

	t	***df***	***p***	***d***
**Grain size**	2.65	208	0.009**	0.366

The *p* value is far less than 0.05, confirming the significant difference between the control and target. Thus, the 4-F-PEAFa additive can improve the grain size of the as-crystalized perovskite.

Note S3. Fitting method of TRPL data.

To fit the TRPL data of the thin films on quartz (Figure 2D), we use the simplified continuity equation without diffusion:

$$\frac{\partial n(t)}{\partial t} = G - k_1 n - k_2 n^2 \tag{S5}$$

where n is the photogenerated charge carrier density, G is the generation rate and k_1 and k_2 are the mono- and bimolecular recombination constants, respectively. G is simulated as a Gaussian laser pulse with a pulse width equal to the time resolution, which is 4 ns in this case (solved using the SciPy package in Python). The solution resembles a power law (i.e., n_{cont}(t)). We then correct this solution with an additional early-time decay as:

$$n_{tot}(t) = A \cdot n_{cont}(t)^2 + (1 - A) \cdot e^{-t/\tau_e} \tag{S6}$$

where A is a scaling factor and τ_e is the lifetime of an early-time effect. This method helps to improve the fittings by estimating early-time effects such as diffusion, without specifically modeling them. For the fluence-dependent TRPL fits, we set τ_e and k_2 to be global variables. The extracted values are summarized below (Note that the simplified model reaches its limit at the highest two fluences measured, as seen from the slightly diverging fit at early times in Figure 2D).

	k_1 (× 10^4 s^{-1})	k_2 (× 10^{-10} cm^3 s^{-1})	τ_e (ns)
Control	9.4	8	1
Target	4.7	8	1

Note S4. Calculation of mobility from the TPC measurements.

To estimate the mobility from the TPC decays, we first calculate the photoconductivity from the photogenerated voltage (V_R) measured with an oscilloscope defined as follows:

$$\sigma_{\mathrm{Photo}} = \left(\frac{V_R}{R_R(V_{\mathrm{App}} - V_R)}\right)\left(\frac{s}{l \times t}\right) \quad \text{(S7)}$$

where R_R is the resistor through which V_R is measured (1000 Ohm), V_{App} is the applied voltage to the sample (~ 3V). s, l and t are the spacing (300 μm), length of the channel between the electrodes (~30 mm), and the film thickness (~500 nm), respectively. We then estimate the sum mobility $\Sigma\mu$ by using the following equation:

$$\sigma_{\mathrm{Photo}}(\mathrm{t}) = q\phi n(t)\Sigma\mu \quad \text{(S8)}$$

where q is the elementary charge, n(t) is the charge carrier density and ϕ is the free carrier fraction. The latter is corrected for early-time recombination and exciton formation at higher fluences as described in more detail in a recent study [3]. As a first approximation, we estimate the mobility at t=0, where n(t) is the excitation density as obtained from the absorption properties of our sample and the laser fluence.

Note S5. Calculation of QFLS.

The QFLS can be calculated from PLQY with the following equation:[47]

$$\mathrm{QFLS} = \frac{k_B T}{q} \ln\left(\frac{J_\mathrm{G}}{J_{0,\mathrm{rad}}} \cdot \mathrm{PLQY}\right) \tag{S9}$$

where J_G is the generation current under illumination, approximated to the short circuit current J_sc in this case. $J_{0,\mathrm{rad}}$ is dark radiative current and ϕ_BB can be calculated with the following equation:

$$J_{0,\mathrm{rad}} = q \int \mathrm{EQE_{PV}(E)}\phi_\mathrm{BB}(\mathrm{E})\mathrm{dE} \tag{S10}$$

$$\phi_\mathrm{BB} = \frac{1}{4\pi^2\hbar^3c^2}\frac{\mathrm{E}^2}{\exp\left(\frac{\mathrm{E}}{\mathrm{k_B T}}\right)-1} \tag{S11}$$

in which q is the elementary charge, ħ is Planck's constant, k_B is Boltzmann constant and T is temperature. Assuming that the perovskite solar cell is at 300 K in thermal equilibrium with its environment, the $J_{0,\mathrm{rad}}$ is calculated to be 7.65 × 10^{-20} A m^{-2} as shown in Figure S22.

Note S6. Calculation of ΔV_{nrad}.

The non-radiative loss (ΔV_{nrad}) can be calculated with the following equation:

$$\Delta V_{\mathrm{nrad}} = \mathrm{QFLS}_{\mathrm{rad}} - \mathrm{QFLS} \quad \text{(S12)}$$

where $\mathrm{QFLS}_{\mathrm{rad}}$ is the radiative QFLS ($\mathrm{EQE}_{\mathrm{EL}}$=1), defined as the following equation:

$$\mathrm{QFLS}_{\mathrm{rad}} = \frac{k_B T}{q} \ln\left(\frac{J_{\mathrm{G}}}{J_{0,\mathrm{rad}}} \cdot 1\right) \quad \text{(S13)}$$

Combing equation S13 with S9, we can find:

$$\mathrm{QFLS}_{\mathrm{rad}} = \mathrm{QFLS} - \frac{k_B T}{q} \ln(\mathrm{PLQY}) \quad \text{(S14)}$$

Thus, considering the PLQY less than 1, the $\mathrm{QFLS}_{\mathrm{rad}}$ indicates the maximum for the splitting of the quasi-Fermi levels.

Note S7. Calculation of FF losses.

Assuming that the solar cell behaves as an ideal diode without series resistance (R_s) and shunt resistance (R_{sh}), a maximum fill factor (FF_{max}) can be obtained from the open-circuit voltage V_{OC} and the ideality factor n_{id}:[48]

$$FF_{max} = \frac{v_{oc} - \ln(v_{oc}+0.72)}{v_{oc}+1} \tag{S15}$$

$$v_{oc} = V_{oc}\frac{q}{n_{id}k_BT} \tag{S16}$$

n_{id} is obtained from the slope fitting in Figure 4D.

From the data of Figure S23, The FF_{max} of control and target is 0.86 and 0.89, separately.

After considering R_s, R_{sh}, the FF can be calculated as follows:

$$FF_s = FF_0(1 - 1.1r_s) + \frac{r_s^2}{5.4} \tag{S17}$$

$$FF = FF_s - \frac{(v_{oc}+0.72)FF_s^2}{v_{oc}r_{sh}} = FF_s(1 - \frac{(v_{oc}+0.72)FF_s}{v_{oc}r_{sh}}) \tag{S18}$$

r_s and r_{sh} is the normalized R_s and R_{sh}, calculated by the characteristic resistance (R_{ch}) :

$$R_{ch} = \frac{V_{oc}}{I_{sc}} \tag{S19}$$

$$r_s = \frac{R_s}{R_{ch}} \tag{S20}$$

$$r_{sh} = \frac{R_{sh}}{R_{ch}} \tag{S21}$$

Compared to the control, the target device showed a decreased R_s from 42.53 to 16.76 Ohm (or 3.57 to 1.41 Ohm·cm^2) and increased R_{sh} from 81,428 to 126,214 Ohm (or 6,840 to 10,602 Ohm·cm^2). The calculated FF is 0.79 and 0.86 for the control and target devices, consistent with the measured values in Figure S31.

References

1. Li F, Deng X, Qi F *et al.* Regulating surface termination for efficient inverted perovskite solar cells with greater than 23% efficiency. *J Am Chem Soc* 2020;**142**:20134–42.
2. Lian Q, Wang P, Wang G *et al.* Doping free and amorphous NiO_x film via UV irradiation for efficient inverted perovskite solar cells. *Adv Sci* 2022;**9**:2201543.
3. Lim J, Kober-Czerny M, Lin Y-H *et al.* Long-range charge carrier mobility in metal halide perovskite thin-films and single crystals via transient photo-conductivity. *Nat Commun* 2022;**13**:4201.
4. Shangshang C, Xuezeng D, Shuang X *et al.* Stabilizing perovskite-substrate interfaces for high-performance perovskite modules. *Science* 2021;**373**:902–7.
5. Ashiotis G, Deschildre A, Nawaz Z *et al.* The fast azimuthal integration Python library: pyFAI. *J Appl Crystallogr* 2015;**48**:510–9.
6. Momma K, Izumi F. VESTA 3 for three-dimensional visualization of crystal, volumetric and morphology data. *J Appl Crystallogr* 2011;**44**:1272–6.
7. Nandi P, Giri C, Swain D *et al.* Room temperature growth of $CH_3NH_3PbCl_3$ single crystals by solvent evaporation method. *CrystEngComm* 2019;**21**:656–61.
8. Oner SM, Sezen E, Yordanli MS *et al.* Surface defect formation and passivation in formamidinium lead triiodide ($FAPbI_3$) perovskite solar cell absorbers. *J Phys Chem Lett* 2022;**13**:324–30.
9. Al-Ashouri A, Köhnen E, Li B *et al.* Monolithic perovskite/silicon tandem solar cell with > 29% efficiency by enhanced hole extraction. *Science* 2020;**370**:1300–1309.
10. Li B, Zhang C, Gao D *et al.* Suppressing oxidation at Perovskite–NiO interface for efficient and stable tin perovskite solar cells. *Adv Mater* 2024;**36**:2309768.
11. Li Z, Yang M, Park J-S *et al.* Stabilizing perovskite structures by tuning tolerance factor: formation of formamidinium and cesium lead iodide solid-state alloys. *Chem Mater* 2015;**28**:284–92.
12. Kim JY, Lee J-W, Jung HS *et al.* High-efficiency perovskite solar cells. *Chem Rev* 2020;**120**:7867–918.
13. Kieslich G, Sun S, Cheetham AK. Solid-state principles applied to organic–inorganic perovskites: new tricks for an old dog. *Chem Sci* 2014;**5**:4712–5.
14. Fu Y, Rea MT, Chen J *et al.* Selective stabilization and photophysical properties of metastable perovskite polymorphs of $CsPbI_3$ in thin films. *Chem Mater* 2017;**29**:8385–94.
15. Niu G, Guo X, Wang L. Review of recent progress in chemical stability of perovskite solar cells. *J Mater Chem A* 2015;**3**:8970–80.
16. Jin-Wook L, Shaun T, Il SS *et al.* Rethinking the A cation in halide perovskites. *Science* 2022;**375**:eabj1186.
17. Zheng X, Li Z, Zhang Y *et al.* Co-deposition of hole-selective contact and absorber for improving the processability of perovskite solar cells. *Nat Energy* 2023; **8**:462–472.
18. Rühle S. Tabulated values of the Shockley–Queisser limit for single junction solar cells. *Sol Energy* 2016;**130**:139–47.
19. Li Z, Li B, Wu X *et al.* Organometallic-functionalized interfaces for highly efficient inverted perovskite solar cells. *Science* 2022;**376**:416–20.
20. Li G, Su Z, Canil L *et al.* Highly efficient p-i-n perovskite solar cells that endure temperature variations. *Science* 2023;**379**:399–403.
21. Jiang Q, Tong J, Xian Y *et al.* Surface reaction for efficient and stable inverted perovskite solar cells. *Nature* 2022; **611**:278–283.
22. Peng W, Mao K, Cai F *et al.* Reducing nonradiative recombination in perovskite

solar cells with a porous insulator contact. *Science* 2023;**379**:683–90.
23. Li F, Deng X, Shi Z *et al.* Hydrogen-bond-bridged intermediate for perovskite solar cells with enhanced efficiency and stability. *Nat Photonics* 2023;**17**:478–484.
24. Tan Q, Li Z, Luo G *et al.* Inverted perovskite solar cells using dimethylacridine-based dopants. *Nature* 2023;**620**:545–551.
25. Park SM, Wei M, Lempesis N *et al.* Low-loss contacts on textured substrates for inverted perovskite solar cells. *Nature* 20233; **624**: 289–294.
26. Liu C, Yang Y, Chen H *et al.* Bimolecularly passivated interface enables efficient and stable inverted perovskite solar cells. *Science* 2023;**382**:810–5.
27. Yu S, Xiong Z, Zhou H *et al.* Homogenized NiO_x nanoparticles for improved hole transport in inverted perovskite solar cells. *Science* 2023;**382**:1399–404.
28. Li Z, Sun X, Zheng X *et al.* Stabilized hole-selective layer for high-performance inverted p-i-n perovskite solar cells. *Science* 2023;**382**:284–9.
29. Liang Z, Zhang Y, Xu H *et al.* Out-of-plane cations homogenise perovskite composition for solar cells. *Nature* 2023;**624**:557–563.
30. Zheng Y, Li Y, Zhuang R *et al.* Towards 26% efficiency in inverted perovskite solar cells via interfacial flipped band bending and suppressed deep-level traps. *Energy Environ Sci* 2024;**17**:1153-1162.
31. Li J, Liang H, Xiao C *et al.* Enhancing the efficiency and longevity of inverted perovskite solar cells with antimony-doped tin oxides. *Nat Energy* 2024;**9**:308–315.
32. Chen P, Xiao Y, Hu J *et al.* Multifunctional ytterbium oxide buffer for perovskite solar cells. *Nature* 2024;**625**:516–22.
33. Azmi R, Utomo DS, Vishal B *et al.* Double-side 2-dimensional/3-dimensional heterojunctions for inverted perovskite solar cells. *Nature* 2024;**628**:93–98.
34. Zhu P, Wang D, Zhang Y *et al.* Aqueous synthesis of perovskite precursors for highly efficient perovskite solar cells. *Science* 2024;**383**:524–31.
35. Chen H, Liu C, Xu J *et al.* Improved charge extraction in inverted perovskite solar cells with dual-site-binding ligands. *Science* 2024;**384**:189–93.
36. Liu S, Li J, Xiao W *et al.* Buried interface molecular hybrid for inverted perovskite solar cells. *Nature* 2024; **632**:536–542.
37. Li S, Xiao Y, Su R *et al.* Coherent growth of high-Miller-index facets enhances perovskite solar cells. *Nature* 2024;**635**:74–881.
38. Zhao K, Liu Q, Yao L *et al.* peri-Fused polyaromatic molecular contacts for perovskite solar cells. *Nature* 2024;**632**:301–6.
39. Wu J, Torresi L, Hu M *et al.* Inverse design workflow discovers hole-transport materials tailored for perovskite solar cells. *Science* 2024;**386**:1256–64.
40. Dong B, Wei M, Li Y *et al.* Self-assembled bilayer for perovskite solar cells with improved tolerance against thermal stresses. *Nat Energy* 2025, DOI: 10.1038/s41560-024-01689-2.
41. Zai H, Yang P, Su J *et al.* Wafer-scale monolayer MoS2 film integration for stable, efficient perovskite solar cells. *Science* 2025;**387**:186–92.
42. Kresse G, Furthmüller J. Efficient iterative schemes for ab initio total-energy calculations using a plane-wave basis set. *Phys Rev B* 1996;**54**:11169–86.
43. Perdew JP, Burke K, Ernzerhof M. Generalized gradient approximation made simple. *Phys Rev Lett* 1997;**78**:1396.
44. Grimme S, Antony J, Ehrlich S *et al.* A consistent and accurate ab initio parametrization of density functional dispersion correction (DFT-D) for the 94 elements H-Pu. *J Chem Phys* 2010;**132**:154104.
45. Kresse G, Joubert D. From ultrasoft pseudopotentials to the projector augmented-wave method. *Phys Rev B* 1999;**59**:1758–75.

46. Frisch MJ, Trucks GW, Schlegel HB *et al.* G16_C01. 2016:Gaussian 16, Revision A.03, Gaussian, Inc., Wallin.
47. Caprioglio P, Stolterfoht M, Wolff CM *et al.* On the Relation between the Open-Circuit Voltage and Quasi-Fermi Level Splitting in Efficient Perovskite Solar Cells. *Adv Energy Mater* 2019;**9**:1901631.
48. Green MA. *Solar Cells: Operating Principles, Technology, and System Applications*. AA(University of New South Wales, Australia), 1982.
